# BAC-pool sequencing and analysis confirms growth-associated QTLs in the Asian seabass genome

**DOI:** 10.1038/srep36647

**Published:** 2016-11-08

**Authors:** Xueyan Shen, Si Yan Ngoh, Natascha May Thevasagayam, Sai Rama Sridatta Prakki, Pranjali Bhandare, Andy Wee Kiat Tan, Gui Quan Tan, Siddharth Singh, Norman Chun Han Phua, Shubha Vij, László Orbán

**Affiliations:** 1 Reproductive Genomics Group, Temasek Life Sciences Laboratory, 117604 Singapore; 2 Nanyang Technological University, 639798 Singapore; 3 Pacific Biosciences, Menlo Park, CA 94025, USA; 4 Department of Animal Sciences and Animal Husbandry, Georgikon Faculty, University of Pannonia, 8360 Keszthely, Hungary; 5 Centre for Comparative Genomics, Murdoch University, Murdoch 6150, Australia

## Abstract

The Asian seabass is an important marine food fish that has been cultured for several decades in Asia Pacific. However, the lack of a high quality reference genome has hampered efforts to improve its selective breeding. A 3D BAC pool set generated in this study was screened using 22 SSR markers located on linkage group 2 which contains a growth-related QTL region. Seventy-two clones corresponding to 22 FPC contigs were sequenced by Illumina MiSeq technology. We co-assembled the MiSeq-derived scaffolds from each FPC contig with error-corrected PacBio reads, resulting in 187 sequences covering 9.7 Mb. Eleven genes annotated within this region were found to be potentially associated with growth and their tissue-specific expression was investigated. Correlation analysis demonstrated that SNPs in *ctsb, skp1* and *ppp2ca* can be potentially used as markers for selecting fast-growing fingerlings. Conserved syntenies between seabass LG2 and five other teleosts were identified. This study i) provided a 10 Mb targeted genome assembly; ii) demonstrated NGS of BAC pools as a potential approach for mining candidates underlying QTLs of this species; iii) detected eleven genes potentially responsible for growth in the QTL region; and iv) identified useful SNP markers for selective breeding programs of Asian seabass.

The Asian seabass (*Lates calcarifer*, Latidae) is a carnivorous, euryhaline marine teleost widely distributed in the Indo-West Pacific region^1^. It is a high-value aquaculture food fish species widely farmed in South-East Asia and Australia, with increasing aquaculture interest in Europe and North America^2^. We have been working on a marker-assisted selection (MAS) program to improve the Asian seabass growth rate for the past eleven years. The selected seabass individuals have shown a substantial increase in their growth rate in comparison to unselected controls. During this period, a number of advanced genomic tools, among them polymorphic DNA markers, expression microarrays, BAC libraries, BAC-based physical map and genetic linkage maps have been developed for the species^3^,^4^,^5^,^6^.

For the development of elite strains with fast growth, improved flesh quality and/or high disease resistance, the identification of genes responsible for these traits is of high importance^7^,^8^,^9^,^10^. Some significant Quantitative Trait Loci (QTL) for growth traits (body weight, total length and standard length) had been identified on linkage group 2 (LG2) of the Asian seabass genetic linkage map^3^. In addition, multiple QTLs for high n-3 PUFA fatty acid traits in flesh^11^ and for resistance to VNN (Viral Nervous Necrosis Virus)^12^ were also described for the species. However, due to the limited number of markers in the QTL regions, it was impossible to map the QTLs to smaller chromosomal regions without a high density linkage map or the whole genome information.

Despite the fact that next generation sequencing (NGS) technologies have reduced the cost and duration of *de novo* genome projects substantially, their short sequencing reads have made the assembly of complex genomes challenging. Although several dozen fish genomes have been sequenced and assembled during the last few years, most of them are draft assemblies that do not tend to cover well complex genomic regions caused by segmental duplications^13^,^14^. Therefore, when the region of interest harboring gene(s) related to QTLs is confined to unknown/complex segments or if the purpose is to improve existing working draft genome assemblies, alternative approaches that target subgenomic regions have been explored. These involve randomly chosen or MTP-based (minimum tiling path) BACs/FPC (Fingerprinted Contigs) and NGS sequencing. Using MTP as a guide, specific BAC clones representing a prioritized genomic interval are selected, pooled, and used to prepare a sequencing library. The first such application was performed in Atlantic salmon: eight BACs belonging to a MTP covering ca. 1 Mb of the Atlantic salmon genome were pooled, sequenced without barcodes using the 454 technology and assembled^15^. Later, similar studies have been carried out in other fish species and plants^16^,^17^,^18^. Sequencing of pooled, contiguous and overlapping BACs not only decreases the cost, but also reduces the complexity of the template to be sequenced.

In this study, we targeted a region of LG2 of the Asian seabass^3^. This LG was predicted earlier to contain several QTLs associated with increased growth^3^. Based on the existing Asian seabass BAC-based physical map^5^, we performed the sequencing, assembly and annotation of BAC pools covering that portion of LG2 by using Illumina MiSeq technology. This study aims to: (i) test the strategy of sequencing and assembly of small-sized BAC pools by NGS technologies for detecting candidate genes underlying growth-related QTLs of this species; ii) provide potential validation/improvement for a complex segment of the Asian seabass *de novo* whole genome assembly; (iii) identify SNP markers for candidate genes potentially associated with increased growth rate in Asian seabass; (iv) perform comparative analysis between LG2-derived sequences from Asian seabass and their homologs in several sequenced teleost genomes.

## Results

### PCR screening of 3D BAC pools identified 22 MTPs/FPC contigs on LG2

**Figure 1:**
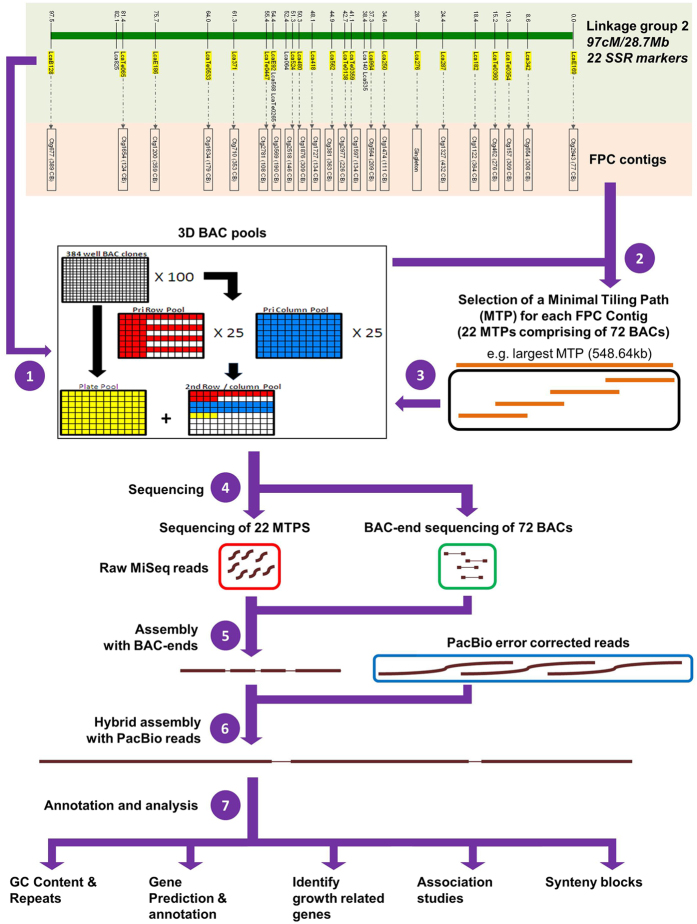
A schematic diagram representing MTP-based BAC pool sequencing, assembly and annotation for a segment of Asian seabass LG2. (1) Generation and screening of 3D BAC pools; (2) 22 MTPs/FPC contigs selection based on a physical map; (**3**) DNA isolation of BACs from selected FPC contigs; (4) Illumina Miseq sequencing of the 22 selected FPC contigs, and Sanger-based BAC-end sequencing of the 72 BACs; 5) *De novo* assembly of all the selected FPC contigs; (6) Miseq and PacBio hybrid assembly; (7) Annotation, synteny analysis and SNP association studies with seabass growth traits (body weight, total length and standard length).

An MTP-based BAC-pool sequencing strategy was established in this study (Fig. 1). Firstly, a three-dimensional (3D) BAC pool set containing 38,400 BAC clones was generated and then PCR-screened for identification of clones containing inserts from the targeted region (see M&M for details). A total of 28 primer pairs (Supplementary Table S1) for the SSR markers located on LG2 were selected to screen the BAC pool set. Following verification, 22 primer pairs identified 86 BAC clones from the 3D BAC pools (Table 1). The average number of BACs amplified per marker was four (range: 1–8) (Table 1). Based on the physical map MTPs/FPC contigs, a total of 72 BAC clones corresponding to 22 MTPs were selected (see M&M for details). The complete list of selected BAC clones, together with their corresponding genetic markers can be found in Table 1.

### Short read sequencing yielded fragmented datasets even after scaffolding

The 22 sequencing libraries (i.e. 22 BAC pools) consisting of 1–7 overlapping BAC clones each were sequenced independently on the Illumina MiSeq platform (Table 1; Supplementary Table S2). Following cleaning and processing, a total of over 3.8 million sequence reads (avg. read length 241.9 bp; range 50–251 bp) were produced. Each BAC was sequenced at over 130x coverage (based on a ~98 kb insert length per BAC). The raw data was submitted to NCBI Short Read Archive (SRA) database (BioProject: SRP063040).

The number of assembled contigs for the 22 BAC pools was between 4 and 75, whereas their average size ranged between 3,338 bp (Ctg1876) and 24,730 bp (Ctg2518). When BAC end sequences (BES) (see M&M for details) were included in the assembly, the former range dropped to 4–65, whereas the latter increased to 3,338–29,676 bp (Table 1; Supplementary Table S3). The final assembly resulted in a total of 5.9 million base pairs, which represented over 84% of the estimated size of the LG2 fragment targeted based on the physical map. We observed that the number of the assembled scaffolds was not consistent with the BAC pooling size in the FPC contigs. For example, in case of Ctg1327 which contained seven partially overlapping BACs, the number of scaffolds in the final assembly was 23; whereas 65 scaffolds were generated for Ctg157 which was formed by only three overlapping BAC clones. The assembled scaffolds were submitted to NCBI database (Genbank Accession No. KT890354-KT890957).

### Hybrid assembly with PacBio reads significantly improved the assembly

As the previous assembly approach yielded unsatisfactory results, error-corrected long reads produced by the Single Molecule Real-Time (SMRT) technology (Pacific Biosystems, from here PacBio; size range ca. 0.5–31.4 kb) from our ongoing Asian seabass Genome Project^19^ were used for scaffolding the MiSeq short-read assemblies of each FPC contig. A total of 32,195 PacBio reads (with the total length of 163.2 Mb) were aligned to the MiSeq-derived scaffolds. The average coverage per BAC clone of the final hybrid assembly was 24-fold of PacBio and 130-fold of MiSeq sequence. The extended assembly was further validated by mapping all the relevant reads and splitting the contigs at regions with zero coverage. We observed that the number of the scaffolds for each FPC contig dropped significantly compared to the MiSeq results (Table 1). For instance, for Ctg710, the scaffold number was reduced from 63 to 19. Similar improvements could be observed for all the 22 FPC contigs (Table 1). The best results were seen for Ctg462: from 46 contigs covering a total of 235.5 kb, a single scaffold of 271.5 kb size was generated after hybrid assembly (Supplementary Fig. S1). The final size of the LG2 segment analyzed from hybrid assembly was 9.73 Mb.

### Sanger sequencing validated NGS results and the hybrid assembly of the 10 Mb region

In addition to mapping back the reads, the validity of the assembly was also evaluated using a BLAST-search of the 22 LG2 genetic markers, 68 BES pairs, and four single BESs. A total of 18 out of the 22 genetic markers were fully aligned to their own FPC contig assemblies (Supplementary Table S4), whereas 61 BES pairs with both ends, five pairs with one end, and the four single BESs were also aligned to their own FPC contig assemblies (Supplementary Table S5). This corresponded to 70 out of 72 (>92%) sequenced BAC clones with an alignment. Furthermore, to evaluate the sequence and assembly accuracy achieved by NGS, we amplified a continuous 9 kb region of the Ctg654_12 by designing 20 primer pairs (Supplementary Table S1) that produced partially overlapping PCR products. They were sequenced by Sanger technology, assembled, and then compared to the NGS-based assembly (see Supplementary file 3 for NGS and Sanger-based sequences and Supplementary file 4 for the comparison). There were only seven nucleotide differences between the Sanger (nucleotides labeled with blue) and the NGS (nucleotides labeled with yellow) assembled sequences. These differences could either be due to sequencing errors or single nucleotide changes.

### Analysis of the BAC-based assembly identified 11 protein-coding sequences potentially associated with growth

A total of 257 genes were detected within the targeted LG2 region (Supplementary Table S6). When these predicted protein sequences were BLAST-searched to derive their gene function^20^, 195/257 (75.9%) were annotated with a gene name and also protein function. Furthermore, from the Blastx results as well as subsequent GO and KEGG pathway analysis (see Materials and Methods for details), we identified eleven genes with potential growth-related function, namely *adcy7, ap2a2, ccne1, csk, ctsb, gas1, megf11, ppp2ca, rab11a, skp1* and protein CYR61-like isoform X1 (Supplementary Table S7). The GO annotation assigned the GO terms of insulin-like growth factor binding, response to fibroblast growth factor, epidermal growth factor receptor signaling pathway, response to growth factor, positive regulation of cell growth and growth factor binding to *ctsb, ppp2ca, csk, ap2a2, rab11* and protein CYR61-like isoform X1, respectively (Supplementary Table S7). Among the eleven genes, *ctsb* is a member of the cathepsin family, which was shown to have major importance during fish post-mortem muscle degradation and softening^21^,^22^. Pathway analysis also showed that *ctsb* is involved in the MHC II pathway, indicating its potential immune related function in Asian seabass. Another four genes *(adcy7, ccne1, ppp2ca* and *skp1)* were involved in the ‘Oocyte meiosis’ pathway^23^, with Igf1 and Igf1r as the most upstream regulators (Supplementary Fig. S2). In addition, both *ppp2ca* and *skp1* were also involved in the TGF-beta signaling pathway^23^ (Supplementary Fig. S3). Interestingly, *ap2a2* and *rab11* were in the ‘Endocytosis’ pathway, with Tgf-beta being the most upstream regulator. *gas1* was involved with the Hedgehog signaling pathway and TGF-beta signaling pathways, whereas *csk* was downstream from *egfr* in the pathway of Epithelial cell signaling in *Helicobacter pylori* infection. In summary, the above analyses indicated that eight out of the 11 genes were mainly associated with four growth-related regulators: Igf1, Igf1r, Egfr and Tgf-beta.

Based on the location of anchored MTPs/FPC contigs by all the 22 genetic markers on LG2^3^, nine out of these 11 genes were shown to be located within the growth-related QTL region (Supplementary Table S7). The remaining two genes (*skp1 and ppp2ca*) were predicted from the assembly of Ctg654 which was screened by the lca342 marker, located adjacent to the QTL region (Supplementary Table S7). Moreover, eight out of nine genes that were located within the QTL region were present in the same assembled scaffold as their own scanning marker (Supplementary Table S7). One of the interesting 11 candidate genes in this QTL region was *ctsb (*cathepsin B), which fell into the same assembled scaffold (ctg1122#Contig [0006] |1-326710|326710) with the genetic marker lca182. In this growth-related QTL region^3^, lca182 and lca287 were the two flanking markers of those three major QTLs for body weight (qBW2-a), total length (qTL2-a) and standard length (qSL2-a) with the highest percentage of phenotypic variance explained (30.2–53.8%).

### Significant differentially expression pattern of eight genes with putative growth function was detected between the tissues of fast- and slow-growing Asian seabass

**Figure 2:**
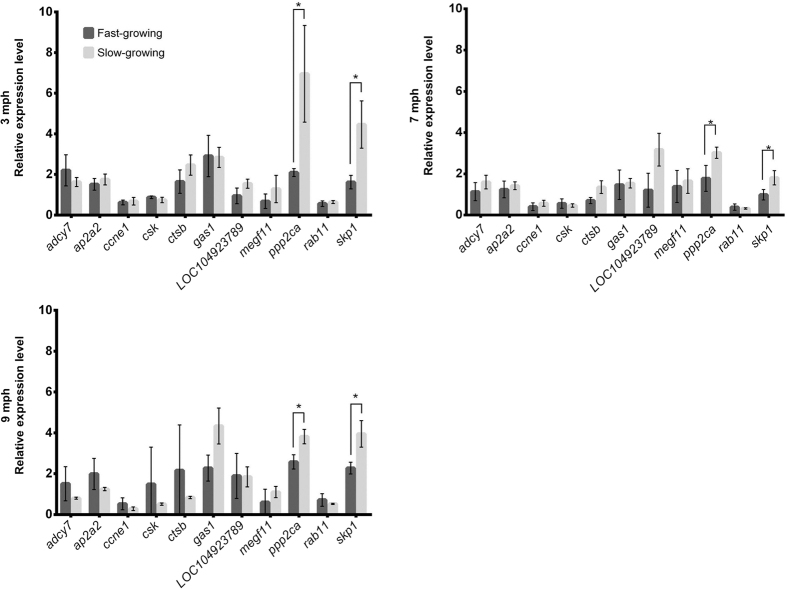
Two genes with potential growth function (*ppp2ca* and *skp1*) showed consistent differential gene expression in the skeletal muscle of Asian seabass at 3, 7 and 9 month post-hatching (mph) of age. Comparison of the relative expression level of 11 genes between the skeletal muscles of fast- and slow-growing Asian seabass individuals at 3, 7 and 9 mph of age. For each pair of bars, the left one (dark grey) indicates fast-growing individual, while the right one (light grey) indicates the slow-growing one. “*” indicates a significant difference (p < 0.05) between the Asian seabass skeletal muscle of fast- and slow-growing controls. *rpl8* and *ef1a* were used as reference genes.

Quantitative RT-PCR was used to study the expression level of the above 11 genes in skeletal muscle, liver and intestine of fast- and slow-growing seabass individuals at 3, 7 and 9 month post-hatching (mph). We observed that the relative expression levels of *skp1* and *ppp2ca* were consistent across all the three developmental stages by having significant downregulation in the muscle of fast-growing individuals compared to slow-growing ones, whereas the expression levels of the remaining genes were not significantly different (Fig. 2).

Besides the muscle, a differential expression pattern of all the eleven genes was also observed in liver and intestine of fast- and slow-growing seabass individuals (Supplementary Table S8). With the exception of *ap2a2, megf11* and *rab11*, the other eight genes were significantly up-regulated in the intestine of slow growing individuals compared to fast-growing ones at 3 mph, whereas only *adcy7* and *ctsb* showed significant higher expression in intestine of fast-growing ones at 7 mph and 9 mph fish, respectively. On the other hand, the expression of *ccne1* together with *csk* and *gas1* was significantly up-regulated in the liver of slow-growing individuals compared to fast-growing ones at 3 mph, while *ppp2ca* were found to be significantly up-regulated only in the liver of fast-growing individuals at 7 mph. Surprisingly, none of these eleven genes showed any significant differences in their expression levels between the liver of fast- and slow-growing individuals at 9 mph (Supplementary Table S8).

### Identification of gene-derived SNPs associated with increased growth rate in Asian seabass

Among the eleven genes, *ctsb* has been shown to be involved in muscle proteolysis and post-mortem degradation in several fish species^22^,^24^,^25^. In addition, the expression level of *skp1* and *ppp2ca* were both significantly down-regulated in skeletal muscle of fast-growing Asian seabass individuals compared to slow-growing ones. Therefore, further association studies were performed for these three genes. They were first sequenced from 20 seabass individuals and their sequence alignment showed a total of 13 polymorphisms, of which three, one and nine were located in *ctsb, skp1* and *ppp2ca*, respectively. Then, we performed a preliminary screen by genotyping 96 fish samples from the same batch (B1) for the above 13 SNP loci. Association analyses were conducted between the genotypes of each SNP and three growth traits (body weight, total length and standard length). Significant associations were detected between all the three traits and genotypes of the SNP in *skp1* (A > T; position: 610 bp) as well as standard length and genotypes of the SNP in *ppp2ca* (A > T; position: 3,439; Table 2). Besides, genotypes of the SNP in *ctsb* (C > T; position: 1,094 bp) showed significant association with seabass body weight (Table 2). In the second phase, all the 13 mutations detected in these three genes were further verified in a larger set of families (batch2, B2) including five families with a total of 570 offspring individuals. In B2, two of the three SNPs identified in *ctsb* showed significant association with the three growth traits. One of them (C > T; position: 1,094 bp) showed that all three growth traits of individuals with CC genotype was significantly higher than those with the CT genotype in B2 (Table 2). Data for the other mutation (A > C; position: 2,461 bp) indicated that individuals with CC genotype had significant association with higher values of all three traits than those with the AC and AA genotype in B2 (Table 2). Among the nine SNPs identified for *ppp2ca*, three of them showed a significant association between the genotype and increased growth traits in B2: 1) A > T (position: 524 bp) with TTs and ATs higher than AAs, except for the body weight with the order of TTs > ATs > AAs; 2) A > T (position: 3,439) with ATs higher than AAs and TTs, and TTs higher than AAs; 3) A > G (position: 3,726 bp) with AGs higher than GGs (Table 2). Frequencies of genotypes and alleles of all these thirteen mutations were also calculated (Table 2). Unfortunately, the SNP locus in *skp1* with significant association between genotype and growth traits in B1 showed no segregation in B2. The details of the above six SNPs exhibiting significant association with growth traits were tabulated in Supplementary Table S9. Besides, Batch 2 was a mix of five families, out of which two have contributed 62.1% and 27.3% of the 570 offspring individuals analyzed, respectively (Supplementary Table S10). We also performed the association studies between genotypes of the above six SNPs and all three growth traits within each of these two major families. The results showed that family-based analysis revealed consistent association patterns with the batch-based analysis (Supplementary Table S11) indicating the associations were not reflecting family structure in this study.

### Homologous chromosome identification and conserved syntenic blocks between Asian seabass LG2 and five other teleost species

**Figure 3:**
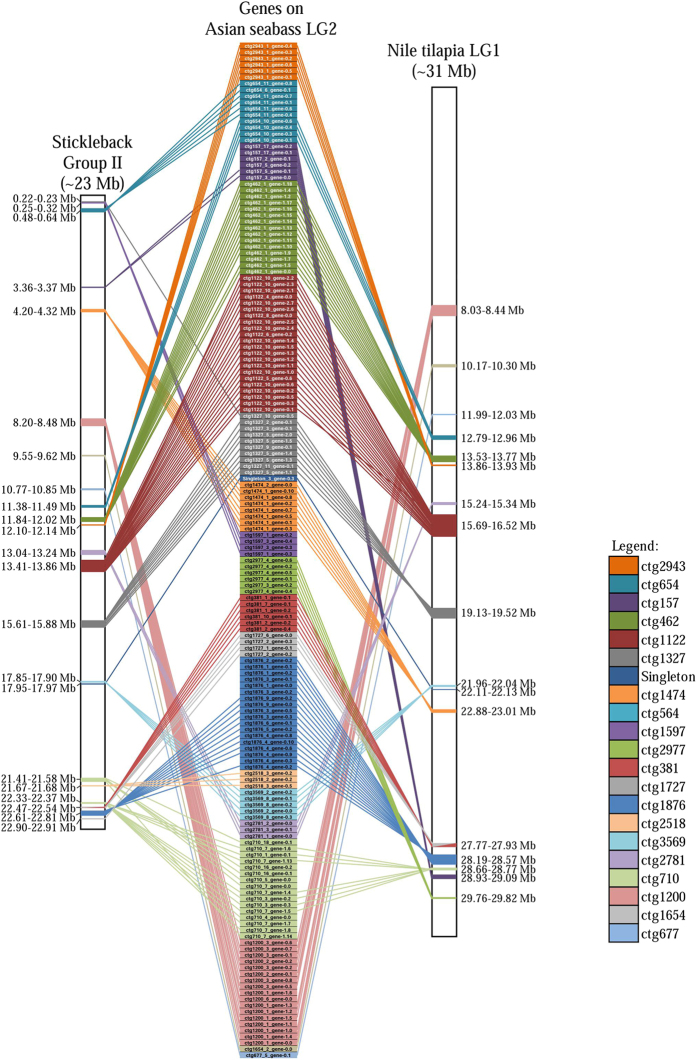
Comparative map of seabass LG2 with Nile tilapia LG1 and three-spined stickleback Group II Asian seabass genome is well conserved at the chromosomal level with those of other teleosts. The Asian seabass LG2 is presented in the center panel, Nile tilapia LG1 and stickleback chromosome group II are on the right and left panel, respectively. For seabass LG2, the gene-associated FPC contigs are indicated by different colours and all the genes names among each FPC contig are given.

The 257 genes identified on seabass LG2 (Supplementary Table S6) were used as queries to search through the genomes of five other sequenced teleost species: the zebrafish, Japanese medaka, three-spined stickleback, spotted green pufferfish and Nile tilapia. A large number of homologous genes (237–248) were found in all five species. The largest number of gene hits per chromosome was found on stickleback Group II (140 hits) although significant number of hits also existed for most of the chromosomes, as well as for unassigned scaffolds in this species (Supplementary Table S12). Similarly, the 257 genes also had a large number of hits on one chromosome of Nile tilapia LG1 (135), medaka chromosome 3 (118), and pufferfish chromosome 5 (117). For zebrafish, two chromosomes, Chr 7 (96) and Chr 18 (47) with significant number of hits were identified (Supplementary Table S12). Conserved synteny analysis was further performed between the seabass LG2 and the homologous chromosome of these five teleost species. A total of 16 conserved syntenies were identified on Group II with 137 genes spanning a total of 29.8 Mb in the stickleback genome. Similarly, 15 conserved syntenies were identified on LG1 of Nile tilapia involving 132 genes. These conserved regions spanned 3.3 Mb of the Nile tilapia genome (Fig. 3, Supplementary Table S13). In addition, conserved syntenic blocks between seabass LG2 and zebrafish Chrs 7 & 18, medaka Chr 3 and pufferfish Chr 5 were also constructed (Supplementary Table S12). Various lengths of conserved syntenies were identified, ranging from 2 –15.7 Mb. Three syntenic blocks containing a total of 62 genes were highly conserved, across all of these fish species (Supplementary Table S13).

## Discussion

### Understanding the potential growth function of genes underlying QTLs in Asian seabass

Generation of fast-growing strains for the rapid improvement of Asian seabass productivity is one of our aims. Through selective breeding, we have obtained large size seabass individuals compared to the unselected controls, which might be explained by additive effects of the potential growth genes^26^. As mentioned above, several genes with a putative role in the enhancement of Asian seabass growth had been identified by our collaborators earlier^7^,^8^,^9^,^10^. In this study, eleven new protein-coding sequences with putative growth function were discovered.

In farmed fish, the freshness and firm texture are considered as the most important quality of the flesh. Among the 11 genes identified here, *ctsb* is of major importance during muscle breakdown and the softening of fish flesh^21^,^22^. As one of the targeted key genes involved in muscle wasting, *ctsb* has been well studied in several fish species during their fasting and refeeding cycle^24^,^25^. In Atlantic halibut and rainbow trout^24^,^25^, *ctsb* showed higher enzyme activities in skeletal muscle of fasted fish than refed ones, which resulted from an increase in mRNA levels of the former. In addition, the expression of *ctsb, ctsd* as well as some genes from the ubiquitin-proteasome (UbP) family in gilthead sea bream skeletal muscle was shown to be coordinately regulated during ontogeny to control muscle growth^22^. In our study, an extremely weak expression of *ctsb* was detected in Asian seabass skeletal muscle. This might indicate a very low enzyme levels, resulting in less muscle wasting and therefore, good fillet tenderization. Further research is needed to elucidate if the expression and/or activity of *ctsb* is correlated with Asian seabass muscle texture. This could provide a practical approach for manipulating the extent of protein breakdown during post-mortem storage of Asian seabass and other related fish species so as to reduce the problems of soft flesh and gaping, which diminish economic value.

In addition to *ctsb, ppp2ca* was also identified with the function of response to fibroblast growth factor in Asian seabass. In human, *ppp2ca* encodes the phosphatase 2A catalytic subunit which is one of the four major Ser/Thr phosphatases and it is implicated in the negative control of cell growth and division with a critical role in embryonic development and human disease^27^. Further, gene *skp1* was shown to be involved with the function of cell growth and death here in Asian seabass. In human and mice, *skp1* encodes a component of SCF (Skp1/Cullin1/F-box) ubiquitin-ligase complex which can bind muscle-specific F-box proteins, for example atrogin-1, and induce muscle atrophy in vertebrates^28^,^29^. Skeletal muscle atrophy is defined as a decrease in muscle mass and it occurs when protein degradation exceeds protein synthesis^30^. Although the function of *ppp2ca* and *skp1* in teleosts is not known, here we observed that their expression was significantly reduced in the muscle of fast-growing seabass individuals in comparison to slower growing controls (Fig. 2). It might be of interest to find out if these two genes mediate a decline in muscle mass through regulation likely of imprinted gene networks in skeletal muscle.

Other genes with potential growth function identified here, include *adcy7* that is related to progesterone-mediated oocyte maturation in European seabass^31^, *ap2a2* that has direct interaction with the epidermal growth factor receptor (*egfr*)^32^, c*cne1* and *csk* that are associated with ovarian cancer tumors and blood pressure in human^33^,^34^. *gas1* is involved in embryonic patterning, inhibits cell proliferation and mediates cell death, and has therefore been considered as a tumor suppressor in human^35^, whereas *rab11* plays an indispensable role in regulating early stages of Drosophila adult muscle development^36^.

According to our knowledge, with the exception of *ctsb*, very little information was available on the function of these genes in teleost muscle growth prior to our study. Here, pathway analyses indicated that eight out of the 11 genes showed association with four major growth-related regulators: Igf1, Igf1r, Egfr and Tgf-beta. In a study on Chilean flounder (*Paralichthys adspersus*), the two Igf molecules were shown to play important roles in the development of muscle and bone-related structures during larval stages^37^. Besides, the Tgf-beta pathway had been shown to regulate the growth, differentiation and metabolism of many cell types, including that of skeletal muscle in mammals^38^. Therefore, to find out how these genes regulate skeletal muscle development and growth in Asian seabass, their specific role must be further investigated by studying the different constituents in the pathways that these genes are involved with (especially the IGF and TGF-beta systems) as well as their interactions in relation to muscle growth.

Significant correlation between markers and traits may indicate the existence of a relationship between them. In such cases, selection breeding based on phenotype can be replaced with the less laborious and more effective genotype-assisted selection^39^. Our analysis showed a significant association between the genotypes at each SNP of *ctsb* (C > T; position: 1,094) and *ppp2ca* (T > A; position: 3,439) and growth traits described earlier in B1 (single family, 96 individuals), was also detected in B2 (five families, 570 individuals). However, an additional SNP from *ctsb* and two more SNPs from *ppp2ca* (with no significant association in B1) showed significant association with the growth traits in B2 only. Unfortunately, the SNP in *skp1* with significant association between genotypes and growth traits in B1, turned out to be monomorphic in B2. The two SNPs showing consistent significant association between the genotypes and growth traits in the two batches may potentially be used as markers for growth traits (especially, when combined more markers) in marker-assisted breeding programs of Asian seabass. However, the other SNPs that showed significant association between genotypes and the growth traits either in B1 or B2 only (see Table 2 for complete list), need to be further validated using a larger number of Asian seabass samples from different populations. A note of caution: as these results were obtained using a relatively small number of animals and families, large-scale verification on additional families would be beneficial to make assertions about population-level association or general application for selective breeding in other stocks.

### Characterization of the 10 Mb assembled genome region, and the observation of highly conserved syntenies of Asian seabass LG2 with five other teleosts

The final assembled genome sequence is about 10 Mb, which accounts for 1.4% of the Asian seabass genome (~700 Mb). The analysis of the assembled sequences allowed us to gain important insights into the euchromatic region of the Asian seabass genome. The sequenced region is relatively AT-rich (59.39%). The total content of the repeats within this 10 Mb region was about 5.6%, and a high proportion of DNA transposons were observed (Supplementary Table S14). Gene prediction and annotation of the assembled sequences resulted in 257 protein-coding genes.

Conserved synteny indicates that homologous genes are co-localized between species, regardless of gene order^40^. Establishing conserved syntenies through comparative interspecies analysis is valuable for genome assembly and annotation as well as for functional and evolutionary genomics studies. Here, through analysis of all the 257 genes, we observed that seabass LG2 is homologous to a single chromosome of stickleback, Nile tilapia, medaka and pufferfish; but two chromosomes of the zebrafish, as was with the catfish LG8^41^. The large number of homologous genes and conserved syntenies among seabass LG2 and the above-mentioned five fish species indicated that the Asian seabass genome is well conserved at the chromosomal level with those of other teleosts. However, substantial chromosomal rearrangement was also observed between seabass LG2 and Nile tilapia Chr 1 as well as stickleback Group II since the separation of the above species from their common ancestors (Fig. 3). These conserved syntenies revealed by identification of gene position and order in other teleost fish species will be potentially useful for genome annotation, as well as functional and evolutionary inference in Asian seabass in the future.

### A recommended sequencing strategy for BAC pools

Using MTP as a guide, BAC clones were selected, pooled without the necessity of barcoding and used to prepare a sequencing library. NGS of BACs has been a viable option for deciphering the sequence of even large and highly repetitive genomes. In our study, for sequencing all of the 22 FPC contigs (1–7 overlapped BACs each), we chose the Illumina MiSeq technology as it provides longer paired-end reads of 2 × 250 bp, and also its lower cost than that of Illumina HiSeq. However, we observed that the assembly yielded high number of contigs/scaffolds for each MTP than expected even with the advantage of paired-end and longer read length. We speculate that this could be due to the presence of several repetitive regions in this genome region, resulting in gaps when conducting *de novo* assembly with short reads. In fact, the high number of assembled contigs or scaffolds from NGS based BAC pool sequencing was also observed in other studies^16^,^42^. For instance, in catfish^16^, twenty-four BAC clones from one MTP were pooled and sequenced. By using different assemblers with different K-values, the 454 and Illumina HiSeq (2 × 100 bp) reads generated from the same BAC pool were assembled separately, which resulted in 279–3,572 contigs. In cotton^42^, five BAC pools with 3–4 BAC clones representing two MTPs were sequenced using 454 technology. A total of 139–301 contigs were generated for each of the BAC pools from 454 assemblies. Compared to the data generated from cotton and catfish, the Illumina MiSeq paired end reads (2 × 250 bp) used in our study generated better assemblies (Supplementary Table S3) for sequencing BAC pools than that of the 454 single reads and also Illumnia HiSeq reads (2 × 100 bp).

The hybrid assembly by combining the dataset generated from different sequencing platforms (such as ABI-Sanger, 454 and Illumnia HiSeq) for a BAC pool had shown that the number of assembled contigs/scaffolds reduced significantly^16^,^42^. In this study, in order to improve the MiSeq assembly, we further utilized self-corrected PacBio long reads for scaffolding the MiSeq short read assemblies of BACs. The PacBio long reads exerted a beneficial effect, as the number of the scaffolds for each FPC contig was significantly or relatively reduced (Table 1; Supplementary Fig. S1). This was due to their spanning of the repetitive regions where DNA complexity seems to cause assemblies based on short reads to terminate. However, the intrinsic shortcoming of raw PacBio long reads is the low accuracy (~15% error rate)^43^. Their self-error correction requires either 80–100X coverage of PacBio sequences or highly accurate short reads^44^. Overall, in order to obtain a high quality assembly from BAC pools based on a limited budget, it is recommended that a combination of Illumina MiSeq short reads (minimum 100x coverage) supplemented with low coverage PacBio longer ones (for both scaffolding and gap filling) be used, especially in those cases when there is a need to sequence through highly complex repetitive genome regions.

## Conclusion

A 10 Mb targeted region of the Asian seabass genome containing important growth-associated QTLs was sequenced and assembled using small BAC pools. The effectiveness of this NGS-based BAC pool approach for identifying candidate genes underlying growth-related QTLs of Asian seabass was demonstrated. A total of 11 genes potentially associated with growth traits were revealed from the QTL region. Association studies suggested that SNPs in *ctsb* and*, ppp2ca* and *skp1* are likely to be useful as markers for selecting fast-growing Asian seabass at fingerling stage. The synteny analysis indicated that Asian seabass genome is well conserved at the chromosomal level with other teleosts; however, extensive signs of inter-chromosomal rearrangements were also observed. As for sequencing BAC pools, the potential benefits of a combination of Illumina MiSeq pair-end short reads with PacBio longer ones are clearly demonstrated. On the long term, this information will be useful for improving biological traits (e.g. growth and meat quality) that are important in Asian seabass aquaculture.

## Methods

Animal experiments were approved by Temasek Life Sciences Laboratory Institutional Animal Care and Use Committee (approval ID: TLL (F)-10-488 003) and performed according to its guidelines.

### Source and 3D pooling of BAC library

A *HindIII* BAC library of the Asian seabass was previously developed by our collaborators^4^. The library consisted of 49,152 clones with an average insert size of 98 kb, representing 6.9 genomic equivalents of the haploid Asian seabass genome (~700 Mb)^45^,^46^.

A physical map of Asian seabass was previously published using SNaPshot HICF FPC technique which containing 38,208 clones from the above BAC library^5^. Out of 38,208 clones, 30,454 were assembled into 2,865 contigs, whereas 4,811 remained singletons. These clones (35,265) cover 4.9- fold of the Asian seabass haploid genome.

In this study, for quick and effective PCR screening of BAC clones with specific DNA sequences, a three-dimensional (3D) BAC pool set containing 16 wells of row pools, 24 wells of column pools and 100 wells of plate pools was constructed as described by Adam-Blondon *et al*.^47^ and Li *et al*.^48^.

### Choice of genetic markers for PCR screening

Based on the genetic linkage map of Asian seabass^3^, twenty-eight SSR markers were selected from Asian seabass LG2 which was shown earlier to contain the growth-related QTLs^3^. Specific primer for each SSR marker (Supplementary Table S1) was used to screen through the above 3D BAC pools in order to identify positive BAC clones. In the first round, PCR was performed with the 100 DNA plate pools, 16 row DNA pools and 24 column DNA pools. Then, an additional round of PCR established the final coordinates in the original BAC library for each positive clone. As more than one positive well was detected in the first round for some products, additional PCR reactions for candidate clones in the original BAC library were necessary for further validation. Six markers (Lca568, LcaTe0265, Lca140, Lca535, Lca064 and Lca825) failed to amplify, even though they produced amplification bands when tested against Asian seabass genomic DNA. Within the fine-mapped QTL region^3^, the two markers in each of the first two pairs (i.e. Lca568-LcaTe0265 and Lca140-Lca535) were located at the same position, respectively, whereas the remaining two markers were outside the fine-mapped QTL region.

### MTP selection, DNA extraction from BAC clones and BAC-end sequencing

Based on the physical map^5^, overlapping BAC clones in a MTP were selected to form small BAC pools. A total of 72 BAC clones corresponding to 22 MTPs (1–7 clones each) were selected, and 22 BAC pools were created finally. Their estimated size was about 5,177 consensus band (CB) units. Based on the recombination rate (3.4 cM/Mb) calculated from the genetic linkage map^3^, the estimated size of the whole LG2 (97 cM) was about 28.7 Mb. Using 1.36 kb/CB unit derived from the physical map^5^, the 22 FPC contigs spanned 7.0 Mb representing 24.4% of LG2 and an estimated 1% of Asian seabass genome (~700 Mb).

Individual preinocules of the selected 72 BACs were grown on 1 ml 1X LB plus 12.5 μg/ml chloramphenicol at 300 rpm, 37 °C, for 17 h. The following day, 30 μl of each BAC clone from the preinocules were added into 50 ml tubes containing 20 ml 1 × LB plus 12.5 μg/ml chloramphenicol, and grown at 37 °C, 300 rpm for 15 h. The bacterial cells were harvested by centrifugation at 6,000 × g for 15 min at 4 °C. Genomic DNA-free BAC DNA extraction was performed using the QIAGEN® midikit (Cat. No. 12145) following manufacturer’s instructions. Final DNA pellets of the 72 BAC clones were resuspended in 200 μl TE (pH 8.0) each. Then equal amount of the individual BAC DNAs from same FPC contig (based on MTP) were combined to form small BAC pool for sequencing. Finally, a total of 22 BAC DNA pools (each containing 1–7 clones) were made from the selected 72 BACs.

End sequences for all the 72 BAC clones were generated by Sanger sequencing using the identical BAC DNA preparations as templates with the universal primers T7 (5′-TAATACGACTCACTATAGGG-3′) and plBRP (5′-CTCGTATGTTGTGTGGAATTGTGAGCC-3′). After quality filtering and vector trimming, 140 high quality BAC end sequences (BES) with an average length of 854 bp were collected. The sequencing success rate was 97.2%. A total of 68 BAC clones were successful sequenced on both ends and the success rate for mate pairs was 94.4%. All the BES are available in (Supplementary file 6).

### Next generation sequencing of pooled BAC clones

Sequencing libraries were made and their sequencing was performed by the Clemson University Genomics Institute (CUGI). Total BAC DNA was purified using standard alkaline lysis methods, followed by digestion with plasmid-safe ATP-Dependent DNase (Epicentre) to reduce host DNA contamination. The purified DNA was quantified by fluorimetry (Life Technologies), and then pooled in equimolar ratios based on the MTP order (FPC contigs) for Nextera (Illumina) library construction following the manufacturer’s recommended procedures. The resulting Nextera libraries were assessed for size distribution on an Agilent Bioanalyzer 2100, which yielded an average fragment size of 475bp. Each of the FPC contig was indexed as a separate library and sequenced on one Illumnia MiSeq (2 × 250 bp) run.

### MiSeq sequence assembly

Sequence assembly was performed by running the datasets from the Nextera paired-end library (PE; 2 × 250bp; 942 bp jump distance) through the assembly workflow. First, reads were trimmed for vector bases, low quality and Illumina adaptors using Trimmomatic v0.30 with the following parameters: “ILLUMINACLIP:illumina.fa:2:40:15 LEADING:3 TRAILING:6 SLIDINGWINDOW:4:15 MINLEN:150”^49^. Next, trimmed reads were kmer (k = 25) normalized to 100X coverage with the normalize_by_kmer_coverage.pl script from the Trinity package v2013_08_14^50^. Preprocessed reads were then assembled using the wgs-assembler v7.0 with default parameters^51^. Sequences of selected genetic markers anchored to the analyzed BACs as well as some BAC-end sequences were used to assign a scaffold to a specific BAC. BAC-end sequences were incorporated into the assemblies using the Phrap software^52^ and manually inspected with Consed^53^. Sequence homology with BES in these regions aided in estimating the efficiency of each assembly process and in predicting the orientation of BACs in the pools.

### Hybrid assembly based on MiSeq and PacBio sequences

Pacific Biosciences (PacBio) RS is a third generation sequencing technology based on single molecule real time (SMRT) sequencing^54^. The technology produces an average read length of ~8–10 kb with the longest one over 20 kb. We took advantage of the availability of a PacBio sequence set (error corrected reads with 21x genome coverage) that was produced for our ongoing Asian seabass Genome Project^19^.

The following steps were performed separately for each FPC contig: PacBio reads that had an overlap with MiSeq assembled contigs were mined based on a mapping of PacBio reads against the MiSeq assembly performed using the CLC Genomics Workbench “Map Reads to Reference” tool (length fraction 0.2, similarity fraction 0.9). Using MIRA, these reads were then co-assembled with MiSeq contigs to which they had mapped. Subsequently, these extended MiSeq-PacBio scaffolds were co-assembled with the remaining MiSeq contigs (to which there were no PacBio reads mapped) using Sequencher (match 99%, min. overlap 20bp). The final hybrid assembly was validated by mapping all the relevant MiSeq and PacBio reads against the scaffolds using the CLC Genomics Workbench “Map Reads to Reference” tool (length fraction 0.95, similarity fraction 0.95).

### Identification and expression analysis of candidate genes with potential growth function

Repeat elements of the assembled sequences were detected using Repeat Masker (version 3.2.7, http://www.repeatmasker.org/). The gene prediction was carried out using MAKER annotation pipeline with the following settings: 1) Augustus gene prediction with “elephant shark” as its model species; 2) sequence homology against thirteen teleost fish species (*Danio rerio, Haplochromis burtoni, Ictalurus punctatus, Lepisosteus oculatus, Maylandia zebra, Neolamprologus brichardi, Oncorhynchus mykiss, Oreochromis niloticus, Oryzias latipes, Pundamilia nyererei, Salmo salar, Takifugu rubripes and Xiphophorus maculates*) proteins downloaded from RefSeq database. Furthermore, potential genes and sequences with growth-related function was derived using the BLAST2GO application^20^,^55^ at www.blast2go.org and the KEGG Automatic Annotation Server (KAAS) (http://www.genome.jp/kegg/kaas/)^56^ with Bi-directional Best Hit (BBH) setting. Firstly, based on the Blastx results and GO terms, we searched for well-known candidate growth-related genes already characterized in other teleosts. Next, the keyword of “growth” was used to extend the search for genes with potential growth-related function based on the gene descriptions and GO terms. These selected genes were further assessed to identify the pathways in which they are involved.

Quantitative real-time PCR (RT-PCR) was performed to analyze the tissue distribution and expression patterns of a selected set of candidate genes with proposed function in growth. A total of 32 Asian seabass individuals from different mass cross populations at 3, 7 and 9 month post-hatching (mph) were used (six fast-growing and six slow-growing individuals each at 3 and 7 mph, while four fast-growing and four slow-growing at 9 mph). Total RNA from muscle, liver and intestine was isolated from these thirty-two individuals. Primers were designed using NCBI Primer-Blast (http://www.ncbi.nlm.nih.gov/tools/primer-blast/) (Supplementary Table S1). Quantitative real-time PCR was performed with ribosomal protein L8 (*rpl8*) and elongation factor-1 alpha (ef1a) as reference genes. Quantification of selected mRNA transcript abundance was performed using solely PCR amplification efficiencies and crossing point (CT) differences^57^.

### SNP detection in genes with potential growth function and association studies of the SNP genotype with Asian seabass growth traits

Two batches of Asian seabass were used for the association studies. The first batch (B1) contained 96 individuals, while batch 2 (B2) was produced by a mass cross of three males and five females, and contained 570 individuals. Multiplex PCR containing nine SSR markers generated for Asian seabass earlier^58^ was utilized to genotype the parents and all the randomly selected offspring. The genotypes were used to carry out the parentage assignment to construct pedigrees using the PAPA v2.0 software^59^. The results showed that all of the 96 individuals from B1 were from one family (only one pair of the brooders has contribution), while the 570 individuals from B2 were assigned to five families with two of them giving major contribution (Supplementary Table S10). The growth traits including body weight (BW), total length (TL) and standard length (SL) at 9 mph from B1, and at 7 mph from B2 were measured.

For SNP identification, nearly complete genomic fragments of three candidate genes (*ctsb, skp1* and *ppp2ca*) with potential function in growth were first screened for nucleotide polymorphisms (SNPs) by using Sanger sequencing in twenty F2 individuals from the above B1 (see Supplementary Table S1 for the primer sequences). The SNPs were genotyped using KBiosciences Competitive AlleleSpecific-PCR SNP genotyping system (KASPar). KASPar assay primers (LGC, Middlesex, UK) were designed using the Kraken™ software using default parameters. Genotyping assays were carried out with the SNPline™ platform (LGC, Middlesex, UK) in standard KASP mix (LGC, Middlesex, UK). KASPar reactions were done following the manufacturer’s protocol (http://www.lgcgroup.com/LGCGroup/media/PDFs/Products/Genotyping/KASP-genotyping-chemistry-User-guide.pdf). The genotypes of SNPs significantly correlated with BW, TL and SL were analyzed using t-test (two genotypes per trait per loci), one way ANOVA (three genotypes per trait per loci) and post ANOVA Tukey’s multiple comparison analysis through software GraphPad Prism 6.

### Comparative analysis of syntenic regions

BLASTX were conducted using all predicted gene-coding sequences to search against ENSEMBL protein databases: zebrafish, medaka, stickleback, pufferfish and Nile tilapia with E-value cutoff of 1E-10, respectively. The homologous chromosomes and gene locations were then identified by BioMart (www.biomart.org) with ENSEMBL gene IDs. The distribution of all predicted LG2 genes on the orthologous chromosomes for these five other fish species were found and tabulated by using our in-house scripts. Homologous chromosomes were identified as the chromosomes with high number of gene hits. Based on SSR markers, the 22 FPC contigs were anchored to the Asian seabass linkage group 2. Conserved syntenies were identified based on genetic positions of SSR markers, BAC end sequences, associated genes of each FPC contigs on the linkage map 2 and model fish chromosomal locations. The putative conserved microsyntenies were identified as segments of model fish chromosomes with a set of adjacent genes that are homologous to a set of adjacent genes in Asian seabass LG2 that are reflected by their colocation within a single FPC contig.

## Additional Information

**Accession codes:** Raw reads of the 22 FPC contigs by MiSeq: NCBI SRA BioProject SRP063040. Assembled scaffolds of the 22 FPC contigs: Genbank accession numbers KT890354-KT890957.

**How to cite this article**: Shen, X. *et al*. BAC-pool sequencing and analysis confirms growth-associated QTLs in the Asian seabass genome. *Sci. Rep.* **6**, 36647; doi: 10.1038/srep36647 (2016).

## Supplementary Material

Supplementary Information

Supplementary Information

Supplementary Information

Supplementary Information

Supplementary Table S3

Supplementary Table S6

Supplementary Table S9

## Figures and Tables

**Table 1:** Correspondence between genetic markers on LG2, positive BAC clones identified from BAC pools, sequenced BAC clones selected based on MTPs/FPC contigs of the Asian seabass physical map and assembled scaffolds by using Miseq alone as well as Miseq-PacBio hybrid assembly.

**Marker**	**Genbank Accession No.**	**Relative position (cM)**	**No. of positive clones detected**	**FPC contig**	**No. of selected BACs on MTP**	**Expected size (kb)**	MiSeq		**MiSeq + PacBio**	
							**No. of scaffolds**	**Assembly size (kb)**	**No. of scaffolds**	**Assembly size (kb)**
LcaE169	HQ233685	0	2	ctg2943	2	100.6	15	120.8	4	164.7
Lca342	DQ290189	8.6	8	ctg654	4	418.9	46	560.3	12	653.4
LcaTe0354	HQ233689	10.3	3	ctg157	3	420.2	65	415.8	25	743.8
LcaTe0360	HQ233690	15.2	2	ctg462	3	375.4	46	235.5	1	271.5
Lca182	DQ290150	18.4	6	ctg1122	5	495.0	18	403.8	11	713.6
Lca287	DQ290155	24.4	3	ctg1327	7	587.5	23	593.4	12	894.4
Lca276	DQ290147	28.7	2	Singleton	1	108.8	4	84.9	3	150.8
Lca250	DQ290127	34.6	4	ctg1474	1	151.0	10	127.6	3	194.8
Lca964	HQ233694	37.3	2	ctg564	3	284.2	36	264.9	11	387.4
LcaTe0359	HQ233696	41.1	3	ctg1597	2	182.2	24	241.4	7	402.6
LcaTe0138	HQ233697	42.7	3	ctg2977	3	307.4	12	189.4	7	378.3
Lca562	HQ233699	44.9	2	ctg381	5	493.7	33	299.1	11	541.4
Lca418	DQ431148	48.1	1	ctg1727	2	182.2	17	145.0	7	360.5
Lca480	HQ233700	50.3	7	ctg1876	5	420.2	58	196.2	13	445.3
Lca524	HQ233701	51.3	2	ctg2518	3	198.6	5	148.4	4	347.2
LcaE92	HQ233705	54.4	2	ctg3569	2	258.4	13	186.4	8	359.7
LcaTe0447	HQ233706	55.4	4	ctg2781	2	146.9	7	254.9	3	246.3
Lca371	DQ290210	61.3	5	ctg710	5	480.1	63	461.1	19	784.2
LcaTe0533	HQ337090	64	7	ctg1634	3	243.4	11	203.1	7	371.5
LcaE186	HQ337091	75.7	8	ctg1200	6	488.2	39	332.0	6	465.9
LcaTe0605	HQ337094	81.4	3	ctg1654	2	168.6	12	146.6	3	369.1
LcaB128	EU072400	97.5	7	ctg677	3	529.0	47	269.8	10	480.5
*Total*	*/*	*/*	*86*	*/*	*72*	*7,040.7*	*604*	*5,880.3*	*187*	*9,726.6*

**Table 2:**
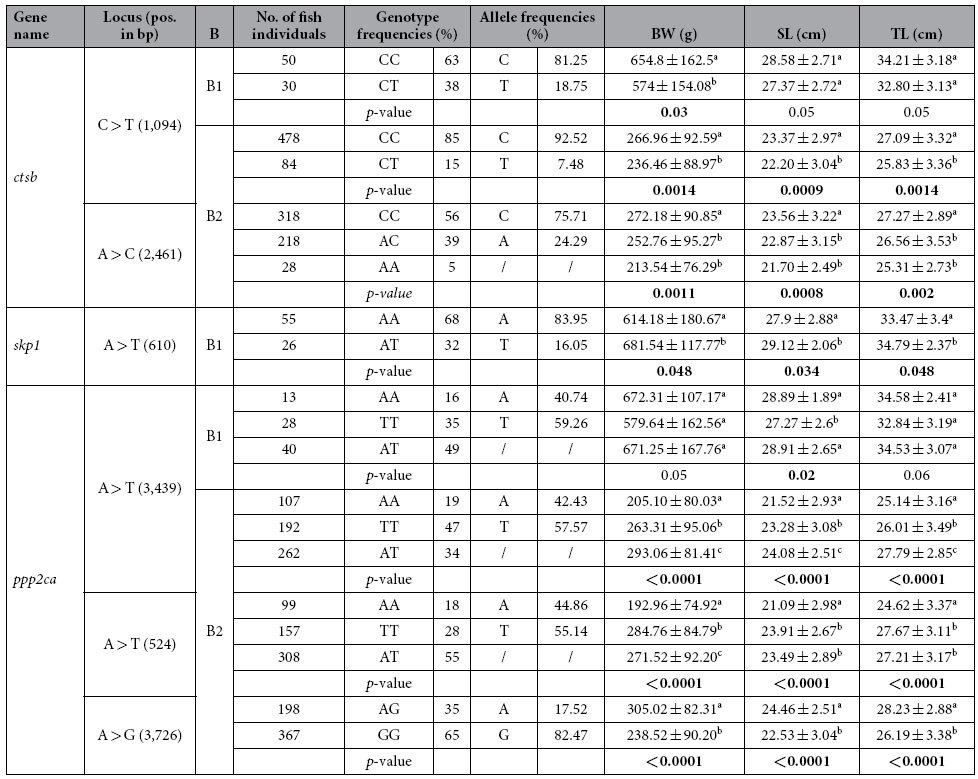
Summary statistics for frequencies of genotypes and alleles for SNPs of *ctsb, skp1* and *ppp2ca* as well as associations between genotypes and growth traits.

Note: 1) B: Batches; B1 includes 96 samples, while B2 includes 570 samples; BW (g), body weight; TL (cm), total length; SL (cm), standard length; 2) The values of body weight and length shown with standard deviation (STDEV); 3) a,b,c, the difference superscript letters within a column indicate a significance (*p* < 0.05); while the same superscript letter within a column means no significant difference (*p* > 0.05); 4) the *p*-value is calculated by t-test and one-way ANOVA, the overall *p*-value is indicated here.
