# Supplementary material for: BAC-pool sequencing and analysis confirms growth-associated QTLs in the Asian seabass genome
Source: Sci Rep. 2016 Nov 8;6:36647. doi: 10.1038/srep36647 (PMC5099610; doi:10.1038/srep36647)
Supplement: Supplementary Information [file srep36647-s4.doc]

**BAC-pool sequencing and analysis confirms growth-associated QTLs in the Asian seabass genome**

*Xueyan Shen^1*^, Si Yan Ngoh^1,2^, Natascha May Thevasagayam^1^, Sai Rama Sridatta Prakki^1^, Pranjali Bhandare^1^, Andy Wee Kiat Tan^1^, Gui Quan Tan^1^, Siddarth Singh^3^, Norman Chun Han Phua^1^, Shubha Vij^1^ and László Orbán^1,4,5*^*

*^1^Reproductive Genomics Group, Temasek Life Sciences Laboratory, Singapore 117604;*

*^2^Nanyang Technological University, Singapore 639798;*

*^3^Pacific Biosciences, Menlo Park, CA 94025, USA;*

*^4^Department of Animal Sciences and Animal Husbandry, Georgikon Faculty, University of Pannonia, 8360 Keszthely, Hungary;*

*^5^Centre for Comparative Genomics, Murdoch University, Murdoch 6150, Australia*

* To whom correspondence should be addressed. Tel: +65/6872-7413 (L.O.); +65/6872-7414 (X.Y.S.); Fax: +65/6872-7007 (L.O. & X.Y.S.). Email: [laszlo@tll.org.sg](mailto:laszlo@tll.org.sg) (L.O.); [xueyan@tll.org.sg](mailto:xueyan@tll.org.sg) (X.Y.S.)

**Supplementary information 1: Supplementary Figures**

**Figure S1.** An example on how the hybrid assembly by Illumnia MiSeq and Pacbio helped in the scaffolding. The original non-overlapping 46 scaffolds of Ctg462 generated by MiSeq were merged into a single scaffold with the help of the error-corrected PacBio long reads.


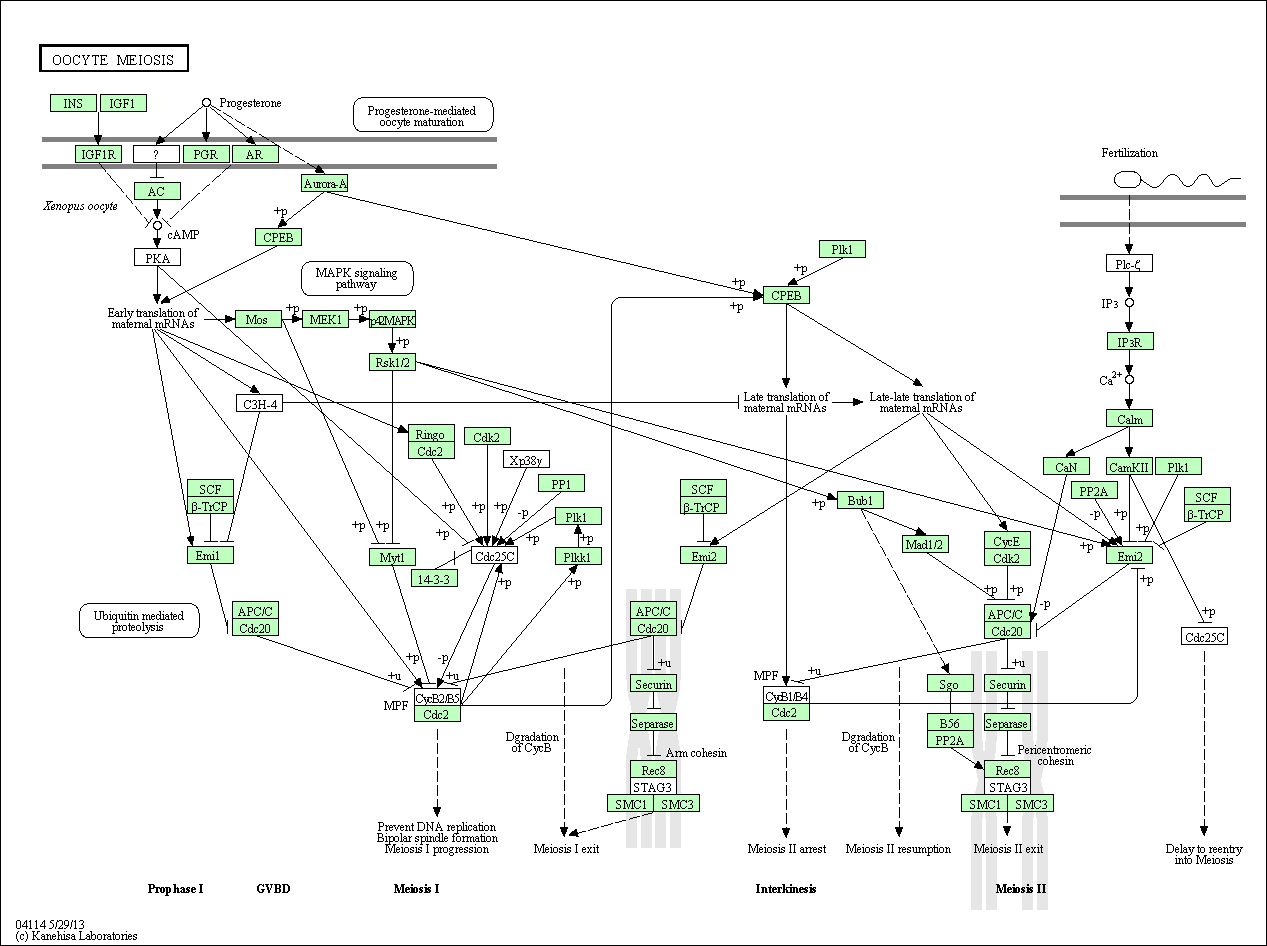


**Figure S2.** Four genes (*adcy7*, *ccne1*, *ppp2ca* and *skp1*) were observed in the Oocyte meiosis pathway with *igf1* and *igf1r* as the most upstream regulating genes.


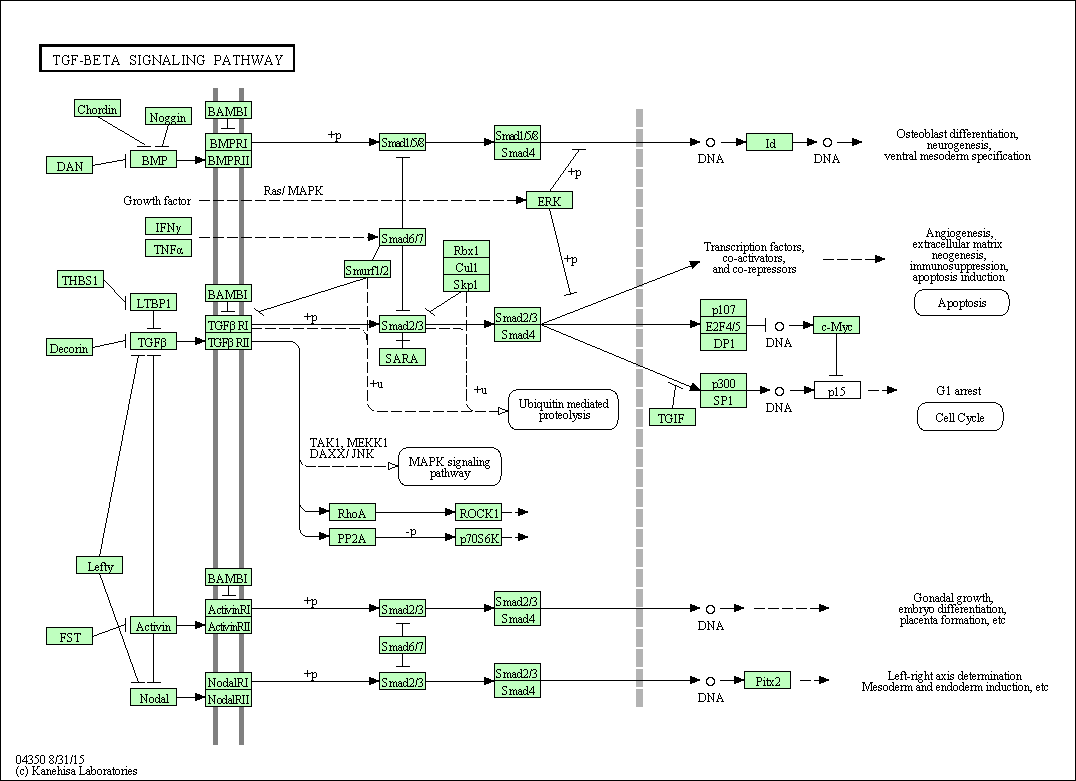


**Figure S3.** Both genes of *ppp2ca* and *skp1* were also involved into the TGF-beta signalling pathway.

**Supplementary information 2: Supplementary Tables**

**Table S1. Primer sequences of SSR markers for BAC pool screening, for the validation of NGS-based assembly, and those of *ctsb*, *ppp2ca* and *skp1* used for SNP identification**

| **Marker/Gene name** | **Forward primer** | **Reverse primer** | **Product size (bp)** |
| --- | --- | --- | --- |
| ***BAC pool screening*** | | | |
| LcaE169 | ATCCCAGCGCACAGCAGTG | GAGGGCCATCTCCGCTCAAG | 182 |
| Lca342 | GAAGCGTGCTGGTGATGTCAAGAG | CTGGCCTGCCTTCATCTGTGAGT | 169-187 |
| LcaTe0354 | AGGGGTTACATGCAAAATAAGTGG | ACAACGTGCTTCCTCCAAATAAAT | 339-343 |
| LcaTe0360 | AAAGCTACAGGACGGTCCATTACA | CTGTCTACTGGCAGCATTGGTTTC | 193-203 |
| Lca182 | ATTTTTGAAGATGTGCCTAATA | CCGAGAAAAGGTTGTTCAC | 216-250 |
| Lca287 | TAATGTTTGGGTATCCGTGTCC | TGACCGAATGAGCTGTTGATAAT | 184-212 |
| Lca276 | GCTGGCATGGCATAACACTTCTTA | GATGCCATGCCAAAGGAAAAAC | 229-245 |
| Lca250 | GCGGATGTATTATGGCAGCAGA | CCCGTGTGGAAGCGAGTTAC | 254-272 |
| Lca964 | AATTGTGCTGTGCGGAGAGT | ACAAATACAAAAGTGACAATAATACA | 143 |
| LcaTe0359 | AGGCTCAGACTGGATGTTACCAAC | TAATGGCAGCCACATACAGGATG | 299-325 |
| LcaTe0138 | ATGGCCCAGGAGGAAGATGA | TTTACTGCCAACCATACTCACTGC | 186 |
| Lca562 | GGACCTAAGCGAGCTAACAGACG | GAGGTAACAGGCCAAGATGTGCT | 358 |
| Lca418 | AAATTGCCCCCAGGTCTGC | TCCTCAATCAGCCACAACTGCT | 216-249 |
| Lca480 | CGCAGCTCAAAGATCTTCAGAGG | TGCTCGGAGTCAAACCCTGTCT | 198 |
| Lca524 | AGGCGTATTACAGGTGATGTGG | GCAGGCCTGTTTTCCTATGC | 200 |
| LcaE92 | TCGCCCTGACCAGAGATAAG | CAAATGTAAGATGGACTGATGTG | 226 |
| LcaTe0447 | TACAGCTCCTGCCCTCCTCAA | GAAGTTTTGGCTGCAACAGTGC | 273-304 |
| Lca371 | GGGCCGGTGATCAGAGACG | GGCAGATCCACATGGACGAGTG | 378-392 |
| LcaTe0533 | CAGCCAAAGAATGATTAAAAGTGA | AGGCCTTATGACCAAGTCTGAGTA | 334-336 |
| LcaE186 | AGGGCCTTCATCGTGCCAACT | CCGGGGATCAAAGGCGAATAA | 186 |
| LcaTe0605 | GTGCTATGGGTTCAGCTGTGTCTC | GCGGATGGTGATAATCAAAGAGG | 342-352 |
| LcaB128 | AGTCGGCCTGTGCAATAAGAT | CAGCAGTTTGGGAATAATGACATA | 262 |
| Lca568 | CGTTAGCGTTTGCAAGAATCTCT | ACACAACCCCACACGCTCTATT | 323 |
| Lca140 | TTCCCCATTTCCTGTCTTTCTGT | GTGTGCACGTGCATGCTTCTAT | 346-372 |
| Lca535 | AACTACGCTGCAGATACAACAAC | CTGCTGCTCTGTCCTTCTCAT | 204 |
| Lca064 | AGGCATATGCACCTCACAAGAGTG | CCCACGGTTTATTTATCTGTCATTATC | 254-290 |
| LcaTe0265 | GTGGCAGCAGGCTTTCA | AGGTCACTGGCTCTTGTTATTT | 245 |
| Lca825 | AAACACATGCATGGTAGCCTGAGG | GTTGTGCTGCTGCCGCTGAG | 105-111 |
| ***NGS-based assembly Ctg_654_12*** | | | |
| Ctg_654_1 | GACAAAAACCTGCGAGGGAG | ATGAAGGGCAAGGGTCAGAG | 936 |
| Ctg_654_2 | ATTGAAGCCATGGAAAGGCG | CACTTTCCAGCCAAAGGAGG | 688 |
| Ctg_654_3 | AGACCGAAAGCAAAGAAGACC | AGCCAGGATACCAGCGAAC | 581 |
| Ctg_654_4 | CCTCCTTTGGCTGGAAAGTG | ATGGAGGAAGCAGCAGAGAG | 944 |
| Ctg_654_5 | GAGCACAATCAACAAGCTACAC | TGTGCTCTAGACACCAGACAG | 531 |
| Ctg_654_6 | TATGGGGGTGTGGAGGAATG | AAACAAACCGCCCTTCTGAC | 829 |
| Ctg_654_7 | TGTGATACCCTTTTCAGGCAC | GAGATCAGCACCACAAGCAG | 520 |
| Ctg_654_8 | CTGCTTGTGGTGCTGATCTC | GACCTGCTACTTCTGGCATC | 506 |
| Ctg_654_9 | CCTCCGCCGTCATCTTTTTC | AAGTGCTATGTCTCCACCCC | 696 |
| Ctg_654_10 | TCGTCATTAGCTGGCAAAGC | AAAGAGAGAGACAGGCCCAC | 736 |
| Ctg_654_11 | TAGTCAGGAAGGGAGGAGGG | GGGGGTTGTTGTGTTCTGTG | 514 |
| Ctg_654_12 | GATCAGTGGGAAACGATGGG | ATGATAACGCACCCTCCTCC | 578 |
| Ctg_654_13 | GCCTGAGCTTTGTCTGTTCC | TGGTGTTCCAAAAGCCGAAG | 725 |
| Ctg_654_14 | TGCCTGAGCTTTGTCTGTTC | CAATTTCCTGCCCACCTCTC | 987 |
| Ctg_654_15 | TGTTCGATTACCTCCCCCTC | TACCTAAACAGCCGCCCTC | 521 |
| Ctg_654_16 | GAGGTGGGCAGGAAATTGTG | CGAGCAAAGTGTGTGAGGAC | 583 |
| Ctg_654_17 | TTTCCCGGCATTTGAACGAG | TGTCTATGGACGGTGACAGG | 961 |
| Ctg_654_18 | ACTCTGTCTTCGGCTTTTGG | TGTCACCGGACATCTCACC | 660 |
| Ctg_654_19 | GACCGTTTACAGGAAGTGCC | GTTCCATGATAGCCGCTTGG | 862 |
| Ctg_654_20 | CAGCTCCCTTGTACCAAGTTC | ACAGGAAGTAGTCTGGGGTG | 528 |
| ***ctsb*** | | | |
| *ctsb_1* | ACCACTGTCCAGTGAGATGG | TAAATGAGACCACCCCACCC | 630 |
| *ctsb_2* | TGTTCTCTCCTTCAGGCTGG | AGACACCTCACACATCCCTG | 723 |
| *ctsb_3* | TGGGGTAAGGAACAGGGATG | TTAGAATCCTGTGCTGCGTG | 795 |
| *ctsb_4* | CAGCTACAGCGAGGACAAAC | CCCACAGCAGATCCATACAC | 661 |
| *ctsb_5* | GTGGACATGCCATCAAGGTC | GGAATGCCTGCCACAATCTC | 632 |
| ***ppp2ca*** | | | |
| *ppp2ca_1* | GACAAAAACCTGCGAGGGAG | ATGAAGGGCAAGGGTCAGAG | 936 |
| *ppp2ca_2* | ATTGAAGCCATGGAAAGGCG | CACTTTCCAGCCAAAGGAGG | 688 |
| *ppp2ca_3* | AGACCGAAAGCAAAGAAGACC | AGCCAGGATACCAGCGAAC | 581 |
| *ppp2ca_4* | CCTCCTTTGGCTGGAAAGTG | ATGGAGGAAGCAGCAGAGAG | 944 |
| *ppp2ca_5* | GAGCACAATCAACAAGCTACAC | TGTGCTCTAGACACCAGACAG | 531 |
| *ppp2ca_6* | TATGGGGGTGTGGAGGAATG | AAACAAACCGCCCTTCTGAC | 829 |
| *ppp2ca_7* | TGTGATACCCTTTTCAGGCAC | GAGATCAGCACCACAAGCAG | 520 |
| *ppp2ca_8* | CTGCTTGTGGTGCTGATCTC | GACCTGCTACTTCTGGCATC | 506 |
| *ppp2ca_9* | CCTCCGCCGTCATCTTTTTC | AAGTGCTATGTCTCCACCCC | 696 |
| *ppp2ca_10* | TCGTCATTAGCTGGCAAAGC | AAAGAGAGAGACAGGCCCAC | 736 |
| *ppp2ca_11* | TAGTCAGGAAGGGAGGAGGG | GGGGGTTGTTGTGTTCTGTG | 514 |
| *ppp2ca_12* | GATCAGTGGGAAACGATGGG | ATGATAACGCACCCTCCTCC | 578 |
| *ppp2ca_13* | GCCTGAGCTTTGTCTGTTCC | TGGTGTTCCAAAAGCCGAAG | 725 |
| *ppp2ca_14* | TGCCTGAGCTTTGTCTGTTC | CAATTTCCTGCCCACCTCTC | 987 |
| *ppp2ca_15* | TGTTCGATTACCTCCCCCTC | TACCTAAACAGCCGCCCTC | 521 |
| *ppp2ca_16* | GAGGTGGGCAGGAAATTGTG | CGAGCAAAGTGTGTGAGGAC | 583 |
| *ppp2ca_17* | TTTCCCGGCATTTGAACGAG | TGTCTATGGACGGTGACAGG | 961 |
| *ppp2ca_18* | ACTCTGTCTTCGGCTTTTGG | TGTCACCGGACATCTCACC | 660 |
| *ppp2ca_19* | GACCGTTTACAGGAAGTGCC | GTTCCATGATAGCCGCTTGG | 862 |
| *ppp2ca_20* | CAGCTCCCTTGTACCAAGTTC | ACAGGAAGTAGTCTGGGGTG | 528 |
| ***skp1*** | | | |
| *skp1_1* | TGGGGAAATCTTCGAGGTGG | TAGAGACGCAGGTAAACCCTC | 853 |
| *skp1_2* | TGATGACCCAGTTCCTCTCC | TGGACAAGGGCAAAAGGAAAC | 865 |
| *skp1_3* | TGTTTCCTTTTGCCCTTGTCC | TACATGTGCACTGGTTTTGGG | 703 |
| *skp1_4* | CCAGACTGGTTTTTCCCCAG | AGACTTGCATGCAGAACACC | 959 |
| *skp1_5* | CCCTGACGTGTTCGAATGTG | TACCTGTGGACGAGATGAGC | 824 |

**Table S2. Preprocessing statistics for all 22 libraries**

| **Library^1^** | **Sequenced Read Pairs** | **Read Pairs Surviving Trim** | **Forward Reads Without Contamination^2^** | **Reverse Reads Without Contamination^2^** | **K-mer Normalized Read Pairs Input  Into Assembler^3^** |
| --- | --- | --- | --- | --- | --- |
| ctg1122 | 513,333 | 408,945 | 194,064 | 194,055 | 76,866 |
| ctg1200 | 376,841 | 306,601 | 71,710 | 71,713 | 60,133 |
| ctg1327 | 697,139 | 557,130 | 138,946 | 139,006 | 86,642 |
| ctg1474 | 119,508 | 99,602 | 37,984 | 37,982 | 25,069 |
| ctg157 | 451,847 | 390,960 | 66,228 | 66,231 | 59,396 |
| ctg1597 | 292,112 | 247,179 | 53,041 | 53,056 | 37,065 |
| ctg1634 | 470,839 | 393,145 | 64,501 | 64,535 | 38,271 |
| ctg1654 | 366,853 | 308,545 | 37,072 | 37,073 | 23,930 |
| ctg1727 | 208,822 | 171,556 | 78,367 | 78,373 | 32,345 |
| ctg1876 | 143,314 | 117,929 | 24,396 | 24,401 | 22,148 |
| ctg2518 | 315,540 | 266,344 | 63,551 | 63,557 | 29,630 |
| ctg2781 | 573,468 | 464,961 | 96,167 | 96,174 | 33,293 |
| ctg2943 | 382,271 | 320,312 | 150,147 | 150,163 | 27,909 |
| ctg2977 | 812,998 | 667,809 | 279,881 | 279,901 | 39,373 |
| ctg3569 | 533,100 | 440,693 | 116,387 | 116,413 | 39,377 |
| ctg381 | 260,198 | 226,543 | 48,479 | 48,495 | 43,666 |
| ctg462 | 153,272 | 128,687 | 33,838 | 33,853 | 32,002 |
| ctg564 | 535,165 | 413,576 | 91,670 | 91,665 | 56,915 |
| ctg654 | 523,187 | 428,575 | 114,705 | 114,717 | 75,810 |
| ctg677 | 285,915 | 243,014 | 46,025 | 46,039 | 37,519 |
| ctg710 | 123,579 | 108,348 | 29,650 | 29,655 | 28,707 |
| Singleton | 308,341 | 243,431 | 111,389 | 111,390 | 21,114 |

*^1^Illumina Nextera 2x250bp paired end reads sequenced on Illumina MiSeq; ^2^E.coli/PhiX/Vector; ^3^K=25 & Coverage = 100X (max)*

**Table S3. MiSeq assembly statistics for 22 libraries (see the separate Excel file)**

**Table S4. Genetic markers were identified in their own FPC contig assemblies**

| **Marker accession no.** | **Marker name** | **Marker length** | **Blast hit** | **Aligned Length** | **Percentage ID** | **Evalue** |
| --- | --- | --- | --- | --- | --- | --- |
| DQ290127 | Lca250 | 297 | ctg1474#ctg1474_scf7180000000118|1-25381|25381 | 297 | 98.99 | 1.00E-148 |
| DQ290147 | Lca276 | 569 | singleton#Singleton_c1|1-77417|77417 | 569 | 97.72 | 0 |
| DQ290150 | Lca182 | 248 | ctg1122#Contig[0006]|1-326710|326710 | 264 | 93.56 | 5.00E-115 |
| DQ290155 | Lca287 | 237 | ctg1327#Contig[0008]|1-134440|134440 | 237 | 83.97 | 5.00E-77 |
| DQ290189 | Lca342 | 407 | ctg654#P05_Ctg654_c1|1-195383|195383 | 407 | 96.56 | 0 |
| DQ290210 | Lca371 | 679 | ctg710#Contig[0004]|54-49813|49760 | 679 | 99.26 | 0 |
| DQ431148 | Lca418 | 574 | ctg1727#Contig[0002]|1-172526|172526 | 565 | 98.05 | 0 |
| EU072400 | LcaB128 | 383 | ctg677#ctg677_c1|1-135418|135418 | 383 | 99.22 | 0 |
| HQ233685 | LcaE169 | 542 | ctg2943#Contig[0001]|1-157472|157472 | 542 | 99.45 | 0 |
| HQ233689 | LcaTe354 | 415 | ctg157#Contig[0005]|1-149533|149533 | 415 | 98.07 | 0 |
| HQ233690 | LcaTe360 | 215 | ctg462#ctg462_c1|1-271457|271457 | 219 | 97.26 | 5.00E-102 |
| HQ233697 | LcaTe0138 | 581 | ctg2977#Contig[0004]|1-199844|199844 | 580 | 98.62 | 0 |
| HQ233699 | Lca562 | 731 | ctg381#Contig[0001]|73564-159053|85490 | 731 | 98.91 | 0 |
| HQ233701 | Lca524 | 280 | ctg2518#Contig[0001]|1-122051|122051 | 282 | 98.23 | 3.00E-137 |
| HQ233705 | LcaE92 | 362 | ctg3569#Contig[0002]|1-38437|38437 | 361 | 100 | 0 |
| HQ337090 | LcaTe0533 | 405 | ctg1634#Contig[0003]|32328-226935|194608 | 405 | 97.53 | 0 |
| HQ337091 | LcaE186 | 308 | ctg1200#Contig[0001]|1-258577|258577 | 307 | 95.58 | 2.00E-146 |
| HQ337094 | LcaTe0605 | 368 | ctg1654#ctg1654_rep_c8|1-36567|36567 | 370 | 98.11 | 0 |

**Table S5. Alignment of BAC-end sequences to the final hybrid assembly by BLAST**

| **S/N** | **BAC-end sequences** | **Blast to final hybrid assembly** |
| --- | --- | --- |
| 1 | Ctg1122_P25-F24_pIBRP_D12_CBP0213 | ctg1122#Contig[0004]|1-68659|68659 |
| 2 | Ctg1122_P25-F24_T7_D12_CBP0213 | ctg1122#Contig[0006]|1-326710|326710 |
| 3 | Ctg1122_P25-H13_pIBRP_G07_CBP0242 | ctg1122#Contig[0004]|1-68659|68659 |
| 4 | Ctg1122_P25-H13_T7_G07_CBP0242 | ctg1122#Contig[0006]|1-326710|326710 |
| 5 | Ctg1122_P39-I07_pIBRP_A04_CBP0383 | ctg1122#Contig[0005]|1-37872|37872 |
| 6 | Ctg1122_P39-I07_T7_A04_CBP0383 | ctg1122#Contig[0006]|1-326710|326710 |
| 7 | Ctg1122_P64-L14_pIBRP_H07_CBP0673 | ctg1122#Contig[0001]|1-31847|31847 |
| 8 | Ctg1122_P64-L14_T7_H07_CBP0673 | ctg1122#Contig[0002]|1-41599|41599 |
| 9 | Ctg1122_P78-H03_pIBRP_G02_CBP1062 | ctg1122#Contig[0006]|1-326710|326710 |
| 10 | Ctg1122_P78-H03_T7_G02_CBP1062 | ctg1122#Contig[0006]|1-326710|326710 |
| 11 | Ctg1200_P34-P07_pIBRP_G04_CBP0334 | ctg1200#ctg1200_c2|1-119858|119858 |
| 12 | Ctg1200_P34-P07_T7_G04_CBP0334 | ctg1200#Contig[0001]|1-258577|258577 |
| 13 | Ctg1200_P68-N17_pIBRP_C09_CBP0794 | ctg1200#Contig[0001]|1-258577|258577 |
| 14 | Ctg1200_P68-N17_T7_C09_CBP0794 | ctg1200#Contig[0001]|1-258577|258577 |
| 15 | Ctg1200_P70-E19_pIBRP_A10_CBP0932 | ctg1200#Contig[0001]|1-258577|258577 |
| 16 | Ctg1200_P70-E19_T7_A10_CBP0932 | ctg1200#ctg1200_c2|1-119858|119858 |
| 17 | ctg1200_P99-K22_plBRP_F11_CBP1273 | ctg1200#Contig[0001]|1-258577|258577 |
| 18 | ctg1200_P99-K22_T7_F11_CBP1273 | ctg1200#ctg1200_c2|1-119858|119858 |
| 19 | Ctg1327_P20-I06_pIBRP_B04_CBP0213 | P37_Ctg1327#Contig[0006]|1-50372|50372 |
| 20 | Ctg1327_P20-I06_T7_B04_CBP0213 | P37_Ctg1327#ctg1327_scf7180000001306|298-21319|21022 |
| 21 | Ctg1327_P33-C07_pIBRP_E04_CBP0321 | P37_Ctg1327#Contig[0008]|1-134440|134440 |
| 22 | Ctg1327_P33-C07_T7_E04_CBP0321 | P37_Ctg1327#Contig[0010]|1-330670|330670 |
| 23 | Ctg1327_P37-E05_pIBRP_A03_CBP0362 | P37_Ctg1327#ctg1327_scf7180000001314|1-16418|16418 |
| 24 | Ctg1327_P37-E05_T7_A03_CBP0362 | P37_Ctg1327#Contig[0010]|1-330670|330670 |
| 25 | Ctg1327_P77-L17_pIBRP_G09_CBP1043 | P37_Ctg1327#Contig[0010]|1-330670|330670 |
| 26 | Ctg1327_P77-L17_T7_G09_CBP1043 | P37_Ctg1327#ctg1327_scf7180000001314|1-16418|16418 |
| 27 | Ctg1327_P94-J23_pIBRP_C12_CBP1223 | P37_Ctg1327#ctg1327_scf7180000001311|1-10906|10906 |
| 28 | Ctg1327_P94-J23_T7_C12_CBP1223 | P37_Ctg1327#Contig[0010]|1-330670|330670 |
| 29 | Ctg1327_P96-G08_pIBRP_F04_CBP1242 | P37_Ctg1327#P37_Ctg1327_v2_rep_c12|1-129729|129729 |
| 30 | Ctg1327_P96-G08_T7_F04_CBP1242 | P37_Ctg1327#P37_Ctg1327_v2_rep_c12|1-129729|129729 |
| 31 | Ctg1327_P97-P15_pIBRP_G08_CBP1254 | P37_Ctg1327#Contig[0010]|1-330670|330670 |
| 32 | Ctg1327_P97-P15_T7_G08_CBP1254 | P37_Ctg1327#Contig[0008]|1-134440|134440 |
| 33 | Ctg1474_P06-F24_pIBRP_D12_CBP0072 | ctg1474#ctg1474_scf7180000000115|1-16816|16816 |
| 34 | Ctg1474_P06-F24_T7_D12_CBP0072 | ctg1474#Contig[0001]|1-152611|152611 |
| 35 | Ctg157_P49-F04_pIBRP_D02_CBP0482 | ctg157#ctg157_c2|1-124309|124309 |
| 36 | Ctg157_P49-F04_T7_D02_CBP0482 | ctg157#Contig[0005]|1-149533|149533 |
| 37 | Ctg157_P87-M07_pIBRP_A06_CBP1154(NG_HR) | ctg157#Contig[0007]|1-34911|34911 |
| 38 | Ctg157_P87-M07_T7_A06_CBP1154 | ctg157#Contig[0004]|1-93724|93724 |
| 39 | Ctg157_P97-H06_pIBRP_H03_CBP1252 | ctg157#ctg157_scf7180000000727|1-11376|11376 |
| 40 | Ctg157_P97-H06_T7_H03_CBP1252 | ctg157#Contig[0005]|1-149533|149533 |
| 41 | Ctg1597_P37-F11_pIBRP_C06_CBP0362 | #N/A |
| 42 | Ctg1597_P37-F11_T7_C06_CBP0362 | ctg1597#Contig[0001]|1-76867|76867 |
| 43 | Ctg1597_P86-E12_pIBRP_B06_CBP1142 | ctg1597#Contig[0002]|1-185628|185628 |
| 44 | Ctg1597_P86-E12_T7_B06_CBP1142 | ctg1597#Contig[0002]|1-185628|185628 |
| 45 | Ctg1634_P91-D11_pIBRP_G06_CBP1191 | ctg1634#Contig[0001]|1-40814|40814 |
| 46 | Ctg1634_P91-D11_T7_G06_CBP1191 | ctg1634#Contig[0003]|1-23822|23822 |
| 47 | Ctg1634_P93-B08_pIBRP_D04_CBP1211 | ctg1634#Contig[0003]|1-23822|23822 |
| 48 | Ctg1634_P93-B08_T7_D04_CBP1211 | ctg1634#Contig[0001]|1-40814|40814 |
| 49 | Ctg1654_P33-D15_pIBRP_G08_CBP0321 | ctg1654#Contig[0003]|1-164049|164049 |
| 50 | Ctg1654_P33-D15_T7_G08_CBP0321 | ctg1654#Contig[0001]|1-168453|168453 |
| 51 | Ctg1654_P91-F21_pIBRP_C11_CBP1192 | ctg1654#Contig[0001]|1-168453|168453 |
| 52 | Ctg1654_P91-F21_T7_C11_CBP1192 | ctg1654#Contig[0003]|1-164049|164049 |
| 53 | Ctg1727_P20-I07_pIBRP_A04_CBP0213(Short_HR) | ctg1727#Contig[0002]|1-172526|172526 |
| 54 | Ctg1727_P20-I07_T7_A04_CBP0213 | ctg1727#ctg1727_scf7180000000485|1-15267|15267 |
| 55 | Ctg1727_P84-M14_pIBRP_B07_CBP1124 | ctg1727#Contig[0001]|1-51487|51487 |
| 56 | Ctg1727_P84-M14_T7_B07_CBP1124 | ctg1727#ctg1727_scf7180000000485|1-15267|15267 |
| 57 | Ctg1876_P18-F01_pIBRP_C01_CBP0192 | ctg1876#Contig[0002]|25073-204343|179271 |
| 58 | Ctg1876_P18-F01_T7_C01_CBP0192 | ctg1876#Contig[0005]|1-24638|24638 |
| 59 | Ctg1876_P38-B14_pIBRP_D07_CBP0371 | ctg1876#Contig[0005]|1-24638|24638 |
| 60 | Ctg1876_P38-B14_T7_D07_CBP0371 | ctg1876#Contig[0002]|25073-204343|179271 |
| 61 | Ctg1876_P85_E06_M13R | ctg1876#Contig[0002]|25073-204343|179271 |
| 62 | Ctg1876_P85_E06_T7 | ctg1876#Contig[0002]|25073-204343|179271 |
| 63 | Ctg1876_P98_K21_M13R | ctg1876#Contig[0002]|25073-204343|179271 |
| 64 | Ctg1876_P98_K21_T7 | ctg1876#Contig[0001]|1-62123|62123 |
| 65 | Ctg2518_P17-L04_pIBRP_H02_CBP0183 | ctg2518#Contig[0001]|1-122051|122051 |
| 66 | Ctg2518_P17-L04_T7_H02_CBP0183 | ctg2518#ctg2518_scf7180000000426|1-3402|3402 |
| 67 | Ctg2518_P40-M22_pIBRP_B11_CBP0394 | ctg2518#Contig[0001]|1-122051|122051 |
| 68 | Ctg2518_P40-M22_T7_B11_CBP0394 | ctg2518#Contig[0001]|1-122051|122051 |
| 69 | Ctg2518_P59-A24_pIBRP_B12_CBP0601 | ctg2518#Contig[0002]|2540-166568|164029 |
| 70 | Ctg2518_P59-A24_T7_B12_CBP0601 | ctg2518#Contig[0001]|1-122051|122051 |
| 71 | Ctg2781_P36-C09_pIBRP_E05_CBP0351 | P63_Ctg2781#Contig[0002]|1-70857|70857 |
| 72 | Ctg2781_P36-C09_T7_E05_CBP0351 | P63_Ctg2781#Contig[0002]|77618-177042|99425 |
| 73 | Ctg2781_P63-K20_pIBRP_F10_CBP0653 | P63_Ctg2781#Contig[0001]|1-76022|76022 |
| 74 | Ctg2781_P63-K20_T7_F10_CBP0653 | P63_Ctg2781#Contig[0002]|1-70857|70857 |
| 75 | Ctg2943_P42-D15_pIBRP_G08_CBP0411 | ctg2943#Contig[0001]|1-157472|157472 |
| 76 | Ctg2943_P42-D15_T7_G08_CBP0411 | ctg2943#Contig[0001]|1-157472|157472 |
| 77 | Ctg2943_P94-P09_pIBRP_G05_CBP1224 | #N/A |
| 78 | Ctg2943_P94-P09_T7_G05_CBP1224 | ctg2943#Contig[0001]|1-157472|157472 |
| 79 | Ctg2977_P68-E13_pIBRP_A07_CBP0792 | ctg2977#Contig[0004]|1-199844|199844 |
| 80 | Ctg2977_P68-E13_T7_A07_CBP0792 | ctg2977#Contig[0004]|1-199844|199844 |
| 81 | Ctg2977_P71-N20_pIBRP_D10_CBP0944 | ctg2977#Contig[0001]|1-32148|32148 |
| 82 | Ctg2977_P71-N20_T7_D10_CBP0944 | ctg2977#Contig[0004]|1-199844|199844 |
| 83 | Ctg2977_P94-N01_pIBRP_C01_CBP1224 | ctg2977#Contig[0004]|1-199844|199844 |
| 84 | Ctg2977_P94-N01_T7_C01_CBP1224 | ctg2977#Contig[0004]|1-199844|199844 |
| 85 | Ctg3569_P54-M24_pIBRP_B12_CBP0544 | ctg3569#ctg3569_c1|1-126224|126224 |
| 86 | Ctg3569_P54-M24_T7_B12_CBP0544 | ctg3569#ctg3569_c1|1-126224|126224 |
| 87 | Ctg3569_P60-O11_pIBRP_E06_CBP0624 | ctg3569#Contig[0001]|1-27746|27746 |
| 88 | Ctg3569_P60-O11_T7_E06_CBP0624 | ctg3569#ctg3569_c1|1-126224|126224 |
| 89 | Ctg381_P05-K05_pIBRP_E03_CBP0063 | ctg381#Contig[0002]|1-121151|121151 |
| 90 | Ctg381_P05-K05_T7_E03_CBP0063 | ctg381#Contig[0001]|1-61818|61818 |
| 91 | Ctg381_P51-L01_pIBRP_G01_CBP0513 | ctg381#Contig[0003]|1-29412|29412 |
| 92 | Ctg381_P51-L01_T7_G01_CBP0513 | ctg381#Contig[0003]|39616-114815|75200 |
| 93 | Ctg381_P51-N13_pIBRP_C07_CBP0514 | #N/A |
| 94 | Ctg381_P51-N13_T7_C07_CBP0514 | #N/A |
| 95 | Ctg381_P70-D18_pIBRP_H09_CBP0931 | ctg381#Contig[0002]|1-121151|121151 |
| 96 | Ctg381_P70-D18_T7_H09_CBP0931 | #N/A |
| 97 | Ctg381_P77-J22_pIBRP_D11_CBP1043 | #N/A |
| 98 | Ctg381_P77-J22_T7_D11_CBP1043 | ctg381#Contig[0001]|73564-159053|85490 |
| 99 | Ctg462_P06-F15_pIBRP_C08_CBP0072 | ctg462#ctg462_c1|1-271457|271457 |
| 100 | Ctg462_P06-F15_T7_C08_CBP0072 | ctg462#ctg462_c1|1-271457|271457 |
| 101 | Ctg462_P62-C04_pIBRP_E02_CBP0641 | #N/A |
| 102 | Ctg462_P62-C04_T7_E02_CBP0641 | #N/A |
| 103 | Ctg462_P99-K13_pIBRP_E07_CBP1273 | ctg462#ctg462_c1|1-271457|271457 |
| 104 | Ctg462_P99-K13_T7_E07_CBP1273 | ctg462#ctg462_c1|1-271457|271457 |
| 105 | Ctg564_P43-I07_pIBRP_A04_CBP0423 | ctg564#Contig[0001]|1-125598|125598 |
| 106 | Ctg564_P43-I07_T7_A04_CBP0423 | ctg564#Contig[0001]|1-125598|125598 |
| 107 | Ctg564_P53-N17_pIBRP_C09_CBP0534 | ctg564#Contig[0002]|1-17309|17309 |
| 108 | Ctg564_P53-N17_T7_C09_CBP0534 | ctg564#Contig[0002]|64843-181309|116467 |
| 109 | Ctg564_P61-D23_pIBRP_G12_CBP0631 | ctg564#Contig[0002]|64843-181309|116467 |
| 110 | Ctg564_P61-D23_T7_G12_CBP0631 | ctg564#Contig[0002]|64843-181309|116467 |
| 111 | Ctg654_P05-B10_pIBRP_D05_CBP0061 | P05_Ctg654#P05_Ctg654_c2|1-138755|138755 |
| 112 | Ctg654_P05-B10_T7_D05_CBP0061 | P05_Ctg654#Contig[0001]|3-6827|6825 |
| 113 | Ctg654_P51-C06_pIBRP_F03_CBP0511 | P05_Ctg654#P05_Ctg654_c3|1-146115|146115 |
| 114 | Ctg654_P51-C06_T7_F03_CBP0511 | P05_Ctg654#P05_Ctg654_c3|1-146115|146115 |
| 115 | Ctg654_P90-M08_pIBRP_B04_CBP1184 | P05_Ctg654#Contig[0002]|1-9635|9635 |
| 116 | Ctg654_P90-M08_T7_B04_CBP1184 | P05_Ctg654#P05_Ctg654_c1|1-195383|195383 |
| 117 | Ctg654_P98-M23_pIBRP_A12_CBP1264 | P05_Ctg654#P05_Ctg654_c2|1-138755|138755 |
| 118 | Ctg654_P98-M23_T7_A12_CBP1264 | P05_Ctg654#P05_Ctg654_c1|1-195383|195383 |
| 119 | Ctg677_P03-K19_pIBRP_E10_CBP0043 | #N/A |
| 120 | Ctg677_P03-K19_T7_E10_CBP0043 | ctg677#ctg677_c1|1-135418|135418 |
| 121 | Ctg677_P35-F22_pIBRP_D11_CBP0342 | ctg677#ctg677_c2|1-104729|104729 |
| 122 | Ctg677_P35-F22_T7_D11_CBP0342 | ctg677#ctg677_c1|1-135418|135418 |
| 123 | Ctg677_P80-023_pIBRP_E12_CBP1084 | ctg677#Contig[0003]|23540-102200|78661 |
| 124 | Ctg677_P80-023_T7_E12_CBP1084 | ctg677#Contig[0003]|1-23153|23153 |
| 125 | Ctg710_P100_H03_M13R | P06_Ctg710#Contig[0005]|88917-351875|262959 |
| 126 | Ctg710_P100_H03_T7 | P06_Ctg710#Contig[0005]|88917-351875|262959 |
| 127 | Ctg710_P17_L08_M13R | P06_Ctg710#Contig[0008]|111-39855|39745 |
| 128 | Ctg710_P17_L08_T7 | P06_Ctg710#Contig[0005]|88917-351875|262959 |
| 129 | Ctg710_p47-J21_pIBRP_H04_C11_CBP0463 | P06_Ctg710#Contig[0005]|88917-351875|262959 |
| 130 | Ctg710_p47-J21_T7_H04_C11_CBP0463 | P06_Ctg710#Contig[0005]|88917-351875|262959 |
| 131 | Ctg710_P6-A07_pIBRP_A04_CBP0071 | P06_Ctg710#Contig[0005]|88917-351875|262959 |
| 132 | Ctg710_P6-A07_T7_A04_CBP0071 | P06_Ctg710#Contig[0005]|88917-351875|262959 |
| 133 | Ctg710_p80-O11_pIBRP_E06_CBP1084 | P06_Ctg710#Contig[0004]|54-49813|49760 |
| 134 | Ctg710_p80-O11_T7_E06_CBP1084 | P06_Ctg710#Contig[0002]|1-32028|32028 |
| 135 | SINGLE_P53-L12_pIBRP_H06_CBP0533 | Singleton#Singleton_c1|1-77417|77417 |
| 136 | SINGLE_P53-L12_T7_H06_CBP0533 | Singleton#Contig[0002]|1-32427|32427 |
| 137 | Ctg1876_P53-G01_T7_E01_CBP0532(LB) | ctg1876#Contig[0002]|25073-204343|179271 |
| 138 | Ctg1634_P43-P03_pIBRP_G02_CBP0424 | ctg1634#Contig[0002]|1-49619|49619 |
| 139 | Ctg1200_P73-M22_pIBRP_B11_CBP0964 | ctg1200#ctg1200_c2|1-119858|119858 |
| 140 | ctg1200_P96-D09_plBRP_G05_CBP1241(HR) | ctg1200#Contig[0001]|1-258577|258577 |

**Table S6. The 257 genes predicted from the assembled regions with their corresponding gene name and GO function (see the separate Excel file)**

**Table S7. The eleven genes potentially associated with growth**

| **Gene symbol** | **Gene name** | **Gene description (by Blastx)** | **GO Term (from BLAST2GO)** | **Associated pathway (from KEGG KAAS)** | **Marker in LG2** | **LG2 marker hit to same assembled scaffold or MTP as the gene** | **Poly-**  **morphism** | **Forward** | **Reverse** |
| --- | --- | --- | --- | --- | --- | --- | --- | --- | --- |
| *skp1* | S-phase kinase-associated protein 1 |  |  | TGF-beta signaling pathway; cell growth and death-Cell cycle pathway | lca342 | Same MTP | Yes | AGGGGATGATGACCCAGTTC | AGGAGGAGGGTCATCTTTGTG |
| *rab11* | Ras-related protein Rab-11 |  | positive regulation of cell growth | Endocytosis  pathway | lca524* | Same scaffold | NTY** | AATACAACAGAGCGCCTTCC | TCATACTCGTCGTCTCTAGTGC |
| *ccne1* | G1/S-specific cyclin-E1 |  |  | cell growth and death-Cell cycle pathway | lcate0138* | Same scaffold | NTY | TGGAGTCTCAGCCCACAAAC | AGCAGGTCGTCTGTCTCTTTC |
| *csk* | C-Src Tyrosine Kinase |  | epidermal growth factor receptor signaling pathway | Epithelial cell signaling in Helicobacter pylori infection pathway | lca250* | Same scaffold | NTY | GGCCTTGAACAGAAAGGAGC | TGTAGTCTCCGACCATCACG |
| *adcy7* | Adenylate Cyclase 7 |  |  | Oocyte meiosis pathway | lca276* | Same scaffold | NTY | TCAGCACCAAGAGGGAACTC | CAACCCACTCGCAGATCAAG |
| *ap2a2* | Adaptor-Related Protein Complex 2, Alpha 2 Subunit |  | response to growth factor | Endocytosis  pathway | lcate0138* | Same scaffold | NTY | GCCGTGTTCATCTCTGACATC | TAGCCGTCTAGAGCCTTGTC |
|
| *ppp2ca* | Serine/threonine-protein phosphatase 2A catalytic subunit alpha isoform |  | response to fibroblast growth factor | TGF-beta signaling pathway; Oocyte meiosis pathway | lca342 | Same MTP | Yes | GCCATGAGAGGAATGTGGTG | GTTCCATGATAGCCGCTTGG |
| *ctsb* | Cathepsin B |  | insulin-like growth factor binding; developmental growth | MHC II pathway | lca182* | Same scaffold | Yes | GTGGCAAGGTCAATGTGGAG | TGAGGGGTAGCCACCATTAC |
| *megf11* | Multiple EGF-Like-Domains 11 | multiple epidermal growth factor-like domains protein 11 |  |  | lca524* | Same scaffold | NTY | GCTCTCAGTGTGCTGAAGTG | GTCCAATGAAGCCGGTTCTG |
| *LOC104923789* | protein CYR61-like isoform X1 |  | growth factor binding; cell growth |  | lca182* | Same MTP | NTY | GGAATGGGTGTGTGACGATG | GCACTGATGTGGTTGGGAAG |
| *gas1* | Growth arrest-specific protein 1 | growth arrest-specific protein 1-like |  | TGF-beta signaling pathways; Hedgehog signaling pathway | lca250* | Same scaffold | NTY | TGTTCAATGGCAGGAAGTGC | CTACCCCATCGCAAACACAG |

* Markers are located within the growth-related QTL region; **NTY - Not tested yet.

**Table S8. The differential gene expression in the skeletal muscle, intestine and liver of Asian seabass individuals at 3, 7 and 9 month post-hatch (mph). The eleven genes listed below were selected based on their potential growth related function.**

| Gene | 3 mph | | | 7 mph | | | 9 mph | | |
| --- | --- | --- | --- | --- | --- | --- | --- | --- | --- |
|  | Intestine | Liver | Muscle | Intestine | Liver | Muscle | Intestine | Liver | Muscle |
| *adcy7* | -4.02*** | -2.47 | 1.29 | 1.29* | -1.18 | -1.56 | 1.61 | 1.77 | 1.72 |
| *ap2a2* | -1.37 | -1.43 | -1.15 | 1.26 | -1.06 | -1.23 | 1.34 | -1.1 | 1.3 |
| *ccne1* | -2.66*** | -2.32** | -1.03 | -1.48 | -2.15 | -1.58 | 1.78 | 1.03 | 1.52 |
| *csk* | -1.74* | -1.76* | 1.22 | -1.07 | -1.2 | 1.04 | 1.85 | 1.12 | 1.14 |
| *ctsb* | -2.13* | -2.08 | -1.53 | 1.03 | -1.39 | -1.77 | 4.80* | 1.75 | 1.19 |
| *gas1* | -1.73** | -4.33* | 1.01 | -1.24 | -1.09 | -1.2 | -1.84 | -1.1 | -1.72 |
| *LOC104923789* | -3.09** | -2.08 | -1.74 | 1.55 | -1.91 | -3.3 | -3.22 | -1.4 | 1.31 |
| *megf11* | -2.51 | -2.22 | -1.78 | 2.3 | 2.23 | -1.2 | -2.53 | 1.29 | 1.11 |
| *ppp2ca* | -1.49** | -1.93 | -2.86* | 1.03 | 1.80* | -1.94* | 2.07 | 1.17 | -1.55* |
| *rab11* | -1.22 | -1.64 | -1.16 | 1.1 | -1.17 | 1.11 | 4.38 | -1.1 | 1.08 |
| *skp1* | -1.63** | -1.76 | -2.58** | -1.02 | -1.17 | -1.90* | 1.7 | 1.07 | -1.78* |

Note: Values represented are fold-changes between fast-growing and slow-growing fishes.
*, *p* < 0.05; **, *p* < 0.01: ***, *p* < 0.001.

**Table S9. The marker and assay sequences for the six SNPs significantly associated with the Asian seabass growth traits (see the separate Excel file)**

**Table S10. The pedigree information of Asian seabass samples in batch 2 used in this study**

| **Female brooder** | **Male brooder** | **No. of Offspring** | **Contribution (%)** |
| --- | --- | --- | --- |
| 1 | A | 354 | 62.11 |
| 2 | B | 156 | 27.36 |
| 3 | A | 25 | 4.38 |
| 4 | B | 23 | 4.03 |
| 5 | C | 12 | 2.10 |

**Table S11. Summary statistics for frequencies of genotypes and alleles for SNPs of *ctsb*, *skp1* and *ppp2ca* as well as associations between genotypes and growth traits of three major families from two batches**

| **Gene name** | **Locus (pos.  in bp)** | **F** | **No. of fish individuals** | **Genotype frequencies (%)** | | **Allele frequencies (%)** | | **BW (g)** | **SL (cm)** | **TL (cm)** |
| --- | --- | --- | --- | --- | --- | --- | --- | --- | --- | --- |
| ***ctsb*** | C>T (1,094) | F1 | 50 | CC | 62.5 | C | 81.25 | 654.8 ± 162.5^a^ | 28.58 ± 2.71^a^ | 34.21 ± 3.18^a^ |
|  |  |  | 30 | CT | 37.5 | T | 18.75 | 574 ± 154.08^b^ | 27.37 ± 2.72^a^ | 32.80 ± 3.13^a^ |
|  |  |  |  | *p-*value |  |  |  | **0.03** | 0.05 | 0.05 |
|  |  | F2 | 241 | CC | 68.9 | C | 84.45 | 291.32 ± 83.10^a^ | 24.11 ± 2.53^a^ | 27.79 ± 2.78^a^ |
|  |  |  | 109 | CT | 31.1 | T | 15.55 | 261.59 ± 91.62^b^ | 23.16 ± 3.04^b^ | 26.79 ± 3.43^b^ |
|  |  |  |  | *p-*value |  |  |  | **0.002** | **0.003** | **0.005** |
|  |  | F3 | 96 | CC | 64.9 | C | 82.45 | 259.42 ± 91.62^a^ | 23.52 ± 2.66^a^ | 27.06 ± 3.08^a^ |
|  |  |  | 52 | CT | 35.1 | T | 17.55 | 227.40 ± 84.870^b^ | 22.16 ± 3.03^b^ | 25.76 ± 3.23^b^ |
|  |  |  |  | *p-*value |  |  |  | **0.01** | **0.02** | **0.03** |
|  | A>C (2,461) | F2 | 213 | CC | 62.5 | C | 75.6 | 284.44 ± 80.40^a^ | 24.22 ± 2.47^a^ | 28.21 ± 2.71^a^ |
|  |  |  | 96 | AC | 28.2 | A | 24.4 | 238.95 ± 93.36^b^ | 22.49 ± 3.22^b^ | 26.09 ± 3.56^b^ |
|  |  |  | 32 | AA | 9.4 | / | / | 226.89 ± 70.32^b^ | 21.07 ± 2.38^b^ | 25.48 ± 3.12^b^ |
|  |  |  |  | *p-value* |  |  |  | **< 0.0001** | **< 0.0001** | **< 0.0001** |
|  |  | F3 | 89 | CC | 57.9 | C | 71.5 | 279.25 ± 85.16^a^ | 24.39 ± 2.18^a^ | 27.39 ± 3.589^a^ |
|  |  |  | 42 | AC | 27.2 | A | 29.5 | 263.56 ± 78.32^b^ | 23.62 ± 3.86^b^ | 25.39 ± 2.65^b^ |
|  |  |  | 23 | AA | 14.9 | / | / | 226.39 ± 82.31^b^ | 22.95 ± 2.69^b^ | 24.87 ± 3.83^b^ |
|  |  |  |  | *p-value* |  |  |  | **0.003** | **0.002** | **0.002** |
| ***skp1*** | A>T (610) | F1 | 55 | AA | 68 | A | 83.95 | 614.18 ± 180.67^a^ | 27.9 ± 2.88^a^ | 33.47 ± 3.4^a^ |
|  |  |  | 26 | AT | 32 | T | 16.05 | 681.54 ± 117.77^b^ | 29.12 ± 2.06^b^ | 34.79 ± 2.37^b^ |
|  |  |  |  | *p-*value |  |  |  | **0.048** | **0.034** | **0.048** |
| ***ppp2ca*** | A>T (3,439) | F1 | 13 | AA | 16 | A | 40.74 | 672.31 ± 107.17^a^ | 28.89 ± 1.89^a^ | 34.58 ± 2.41^a^ |
|  |  |  | 28 | TT | 35 | T | 59.26 | 579.64 ± 162.56^a^ | 27.27 ± 2.6^b^ | 32.84 ± 3.19^a^ |
|  |  |  | 40 | AT | 49 | / | / | 671.25 ± 167.76^a^ | 28.91 ± 2.65^a^ | 34.53 ± 3.07^a^ |
|  |  |  |  | *p-*value |  |  |  | 0.05 | **0.02** | 0.06 |
|  |  | F2 | 50 | AA | 14.1 | A | 31.9 | 216.25 ± 80.75^a^ | 21.85 ± 2.89^a^ | 25.26 ± 3.58^a^ |
|  |  |  | 178 | TT | 50.3 | T | 68.1 | 275.78 ± 92.56^b^ | 23.56 ± 3.73^b^ | 26.89 ± 3.25^b^ |
|  |  |  | 126 | AT | 35.6 | / | / | 290.96 ± 78.85^c^ | 24.98 ± 3.09^c^ | 27.76 ± 2.78^c^ |
|  |  |  |  | *p-*value |  |  |  | **< 0.0001** | **< 0.0001** | **< 0.0001** |
|  |  | F3 | 20 | AA | 13 | A | 29.55 | 196.23 ± 78.15^a^ | 20.98 ± 2.34^a^ | 24.95 ± 2.26^a^ |
|  |  |  | 83 | TT | 53.9 | T | 70.45 | 276.25 ± 87.23^b^ | 23.59 ± 2.78^b^ | 26.38 ± 2.95^b^ |
|  |  |  | 51 | AT | 33.1 | / | / | 298.32 ± 76.35^c^ | 24.68 ± 2.96^c^ | 28.16 ± 3.01^c^ |
|  |  |  |  | *p-*value |  |  |  | **< 0.0001** | **< 0.0001** | **< 0.0001** |
|  | A>T (524) | F2 | 58 | AA | 16.5 | A | 43.35 | 191.05 ± 82.16^a^ | 20.98 ± 3.33^a^ | 24.50 ± 3.77^a^ |
|  |  |  | 105 | TT | 29.8 | T | 56.65 | 286.76 ± 79.92^b^ | 23.85 ± 2.58^b^ | 28.75 ± 2.52^b^ |
|  |  |  | 189 | AT | 53.7 | / | / | 270.69 ± 72.20^c^ | 23.96 ± 3.72^b^ | 28.31 ± 2.87^b^ |
|  |  |  |  | *p-*value |  |  |  | **< 0.0001** | **< 0.0001** | **< 0.0001** |
|  |  | F3 | 29 | AA | 19.3 | A | 47.65 | 191.86 ± 77.85^a^ | 20.89 ± 3.98^a^ | 23.76 ± 2.89^a^ |
|  |  |  | 36 | TT | 24 | T | 52.35 | 278.85 ± 83.46^b^ | 22.71 ± 2.25^b^ | 26.59 ± 3.42^b^ |
|  |  |  | 85 | AT | 56.7 | / | / | 263.39 ± 89.78^c^ | 22.36 ± 2.78^b^ | 26.35 ± 3.86^b^ |
|  |  |  |  | *p-*value |  |  |  | **< 0.0001** | **< 0.0001** | **< 0.0001** |
|  | A>G (3,726) | F2 | 126 | AG | 36.3 | A | 18.15 | 301.44 ± 84.13^a^ | 24.37 ± 2.58^a^ | 28.05 ± 2.85^a^ |
|  |  |  | 221 | GG | 63.7 | G | 81.85 | 278.74 ± 81.23^b^ | 23.57 ± 2.49^b^ | 27.41 ± 2.79^b^ |
|  |  |  |  | *p-*value |  |  |  | **0.01** | **0.02** | **0.04** |
|  |  | F3 | 27 | AG | 17.9 | A | 8.95 | 320.06 ± 92.76^a^ | 24.58 ± 2.91^a^ | 28.36 ± 3.10^a^ |
|  |  |  | 124 | GG | 82.1 | G | 91.05 | 199.77 ± 78.56^b^ | 21.32 ± 3.07^b^ | 24.93 ± 3.37^b^ |
|  |  |  |  | *p-*value |  |  |  | **0.0001** | **0.001** | **0.0007** |

Note: 1) F:families; F1 includes 96 samples from batch 1, while F2 and F3 from batch 2 contains 354 and 156 samples, respectively; BW (g), body weight; TL (cm), total length; SL (cm), standard length; 2) The values of body weight and length shown with standard deviation; 3) a,b,c, the different superscript letters within a column indicate a significance (*p*<0.05); while the same superscript letter within a column means no significant difference (*p*>0.05); 4) the *p*-value is calculated by t-test and one-way ANOVA, the overall *p*-value is indicated here.

**Table S12. Distributions of LG2 genes on orthologous chromosomes of five fish species**

| **Chromosome** | **No. of gene hits** | | | | |
| --- | --- | --- | --- | --- | --- |
|  | **Zebrafish** | **Medaka** | **Stickleback** | **Green-spotted pufferfish** | **Nile tilapia** |
| 1 | 1 | 5 | 4 | 2 | **135** |
| 2 | 3 | 1 | **140** | 0 | 1 |
| 3 | 11 | **118** | 4 | 1 | 0 |
| 4 | 0 | 4 | 1 | 2 | 9 |
| 5 | 13 | 0 | 2 | **117** | 18 |
| 6 | 3 | 18 | 1 | 1 | 5 |
| 7 | **96** | 2 | 6 | 7 | 8 |
| 8 | 2 | 12 | 0 | 7 | 0 |
| 9 | 0 | 6 | 3 | 0 | 0 |
| 10 | 4 | 0 | 1 | 0 | 3 |
| 11 | 2 | 1 | 9 | 0 | 15 |
| 12 | 0 | 4 | 3 | 5 | 8 |
| 13 | 7 | 5 | 14 | 18 | 2 |
| 14 | 1 | 7 | 4 | 3 | 1 |
| 15 | 6 | 0 | 0 | 3 | 0 |
| 16 | 0 | 3 | 1 | 1 | 0 |
| 17 | 15 | 5 | 0 | 2 | 1 |
| 18 | **47** | 2 | 2 | 4 | 2 |
| 19 | 3 | 0 | 14 | 1 | 0 |
| 20 | 3 | 5 | 2 | 0 | 2 |
| 21 | 5 | 2 | 1 | 1 | 0 |
| 22 | 1 | 1 | 0 | 0 | 0 |
| 23 | 3 | 0 | 0 | 0 | 2 |
| 24 | 2 | 4 | 0 | 0 | 0 |
| 25 | 14 | 0 | / | 0 | 0 |
| Unassigned scaffolds | 4 | 35 | 26 | 62 | 36 |
| **Total** | **246** | **240** | **238** | **237** | **248** |

**Table S13. Summary of conserved syntenic blocks between Asian seabass LG2 and chromosomes of five other fish species**

| **Asian seabass physical contigs** | **Stickleback Group II** | | | **Nile tilapia LG1** | | | **Zebrafish Chr 7** | | | | **Zebrafish Chr 18** | | | **Medaka Chr 3** | | | **Green-spotted pufferfish Chr 5** | | |
| --- | --- | --- | --- | --- | --- | --- | --- | --- | --- | --- | --- | --- | --- | --- | --- | --- | --- | --- | --- |
|  | **Synteny block** | **Number of genes**^#^ | **Spanning size (kb)** | **Synteny block** | **Number of genes**^#^ | **Spanning size (kb)** | | **Synteny block** | **Number of genes**^#^ | **Spanning size (kb)** | **Synteny block** | **Number of genes**^#^ | **Spanning size (kb)** | **Synteny block** | **Number of genes**^#^ | **Spanning size (kb)** | **Synteny block** | **Number of genes**^#^ | **Spanning size (kb)** |
| **ctg2943** | * | 6 | 45 | * | 6 | 64 | |  | 0 | 0 | * | 6 | 132 | * | 4 | 39 | * | 5 | 29 |
| **ctg654** | * | 10 | 11,010 | * | 4 | 168 | | * | 13 | 397,53 |  | 0 | 0 | * | 12 | 15,703 | * | 4 | 79 |
| **ctg157** | * | 2 | 9 | * | 6 | 164 | | * | 6 | 731 |  | 0 | 0 |  | 0 | 0 |  | 0 | 0 |
| **ctg462** | * | 14 | 179 | * | 15 | 239 | | * | 2 | 151 | * | 8 | 19,005 | * | 12 | 229 | * | 11 | 145 |
| **ctg1122** | * | 19 | 448 | * | 22 | 828 | |  | 0 | 0 |  | 0 | 0 | * | 19 | 595 | * | 20 | 317 |
| **ctg1327** | * | 10 | 15,655 | * | 9 | 389 | | * | 8 | 421 | * | 5 | 287 | * | 8 | 394 | * | 6 | 11,221 |
| **Singleton** |  | 1 | 20 |  | 1 | 27 | |  | 1 | 44 |  | 0 | 0 |  | 1 | 31 |  | 0 | 0 |
| **ctg1474** | * | 8 | 126 | * | 8 | 135 | |  | 0 | 0 | * | 4 | 4756 | * | 7 | 153 |  | 0 | 0 |
| **ctg564** |  | 0 | 0 |  | 0 | 0 | |  | 0 | 0 |  | 0 | 0 |  | 0 | 0 |  | 0 | 0 |
| **ctg1597** | * | 4 | 103 |  | 0 | 0 | | * | 6 | 327 |  | 0 | 0 | * | 2 | 22 |  | 0 | 0 |
| **ctg2977** |  | 0 | 0 | * | 6 | 62 | | * | 4 | 25 |  | 1 | 8 | * | 5 | 35,150 |  | 0 | 0 |
| **ctg381** | * | 6 | 69 | * | 6 | 156 | | * | 6 | 227 |  | 1 | 2 |  | 0 | 0 | * | 7 | 4,369 |
| **ctg1727** | * | 4 | 436 | * | 3 | 17 | | * | 3 | 54 |  | 0 | 0 |  | 0 | 0 | * | 3 | 263 |
| **ctg1876** | * | 11 | 202 | * | 18 | 374 | | * | 3 | 22,946 | * | 6 | 39,519 |  | 0 | 0 | * | 12 | 82 |
| **ctg2518** | * | 3 | 9 |  | 0 | 0 | | * | 6 | 108 | * | 5 | 8,849 | * | 3 | 84 | * | 6 | 33 |
| **ctg3569** | * | 5 | 55 | * | 5 | 71 | | * | 5 | 106 | * | 5 | 491 | * | 5 | 74 |  | 0 | 0 |
| **ctg2781** | * | 3 | 204 | * | 3 | 98 | |  | 0 | 0 |  | 0 | 0 | * | 3 | 190 | * | 3 | 176 |
| **ctg710** | * | 16 | 967 | * | 7 | 107 | | * | 17 | 26,791 | * | 6 | 6,834 | * | 20 | 2,331 | * | 22 | 244 |
| **ctg1634** |  | 0 | 0 |  | 0 | 0 | |  | 0 | 0 |  | 0 | 0 |  | 0 | 0 |  | 0 | 0 |
| **ctg1200** | * | 16 | 279 | * | 14 | 407 | | * | 14 | 11812 |  | 0 | 0 | * | 15 | 388 | * | 17 | 241 |
| **ctg1654** |  | 1 | 73 |  | 1 | 122 | |  | 1 | 158 |  | 0 | 0 |  | 1 | 77 |  | 1 | 92 |
| **ctg677** |  | 1 | 77 |  | 1 | 45 | |  | 1 | 158 |  | 0 | 0 |  | 1 | 75 |  | 0 | 0 |
| **Total / 22** | **16** | **137(140)** | **29,796** | **15** | **132(135)** | **3,279** | | **13** | **93(96)** | **103,452** | **8** | **45(47)** | **79,873** | **13** | **115(118)** | **55,352** | **12** | **116(117)** | **17,199** |

^#^ Number of genes within synteny blocks; total number of genes are shown in parenthesis.

**Table S14. Repeat elements in the assembled Asian seabass LG2**

| **Elements** | **Number of elements** | **Length occupied (bp)** | **Percentage of sequences** |
| --- | --- | --- | --- |
| **SINEs** | 116 | 11794 | 0.12% |
| **LINEs** | 326 | 87291 | 0.90% |
| L2/CR1/Rex | 269 | 68449 | 0.70% |
| R1/LOA/Jockey | 6 | 398 | 0.00% |
| R2/R4/NeSL | 8 | 2405 | 0.02% |
| RTE/Bov-B | 27 | 12584 | 0.13% |
| L1/CIN4 | 16 | 3455 | 0.04% |
| **LTR elements** | 222 | 35478 | 0.36% |
| BEL/Pao | 13 | 1849 | 0.02% |
| Ty1/Copia | 2 | 3440 | 0.04% |
| Gypsy/DIRS1 | 133 | 24764 | 0.25% |
| Retroviral | 5 | 442 | 0.00% |
| **DNA transposons** | 1072 | 124965 | 1.28% |
| hobo-Activator | 316 | 22011 | 0.23% |
| Tc1-IS630-Pogo | 332 | 65099 | 0.67% |
| PiggyBac | 18 | 1649 | 0.02% |
| Tourist/Harbinger | 18 | 2546 | 0.03% |
| **Unclassified** | 106 | 7123 | 0.07% |
| **Small RNA** | 27 | 2641 | 0.03% |
| **Satellites** | 25 | 2035 | 0.02% |
| **Simple repeats** | 5857 | 230882 | 2.37% |
| **Low complexity** | 786 | 43089 | 0.44% |

**Supplementary information 3:** The full sequence of the Ctg_654_12 hybrid assembled using MiSeq and PacBio reads, and also the sequence assembled by Sanger sequencing (**see the separate FASTA file**)

**Supplementary information 4:** CLUSTAL 2.1 multiple sequence alignment of Ctg654_12_Sanger_assembly and Ctg654_12_NGS_hybrid_assembly

Ctg654_12_Sangerassembly --------------------------------------------------

Ctg654_12_NGS_assembly AAACCTGCGAGGGAGTCGTCTCCTAACCGAAGTTCGCCAACGTTTGGGCA 100

Ctg654_12_Sangerassembly ------ACTGCAGCGACCCCGAAAACACACAGAAATGGACGAAAAGGCGT 44

Ctg654_12_NGS_assembly GAGAAAACTGCAGCGACCCCGAAAACACACAGAAATGGACGAAAAGGCGT 150

********************************************

Ctg654_12_Sangerassembly TTACGAAAGAACTTGATCAGTGGATCGAGCAGCTCAACGAGTGCAAGCAG 94

Ctg654_12_NGS_assembly TTACGAAAGAACTTGATCAGTGGATCGAGCAGCTCAACGAGTGCAAGCAG 200

**************************************************

Ctg654_12_Sangerassembly CTGTCGGAGGGACAAGTGAAAACACTTTGTGAAAAGGTGAGATATACATA 144

Ctg654_12_NGS_assembly CTGTCGGAGGGACAAGTGAAAACACTTTGTGAAAAGGTGAGATATACATA 250

**************************************************

Ctg654_12_Sangerassembly TTTTTAAAATTCACCCCCCCCCCTCCCCAACCCCAAGTAACTGTCAACGT 194

Ctg654_12_NGS_assembly TTTTTAAAATTCACCCCCCCCCCTCCCCAACCCCAAGTAACTGTCAACGT 300

**************************************************

Ctg654_12_Sangerassembly CTTCTCTCTCCTTACCGCCGCCTTCCAGACATTCTTAAAATGGCGTCTAT 244

Ctg654_12_NGS_assembly CTTCTCTCTCCTTACCGCCGCCTTCCAGACATTCTTAAAATGGCGTCTAT 350

**************************************************

Ctg654_12_Sangerassembly AGTGTACATTTCACTGCGCTCTGCGTATATTTGACAATCATTATGAGCGG 294

Ctg654_12_NGS_assembly AGTGTACATTTCACTGCGCTCTGCGTATATTTGACAATCATTATGAGCGG 400

**************************************************

Ctg654_12_Sangerassembly TTAGCTTCTTCTTTTAATAGGACGTTTGAACCGTTGTCCGACAGAAATGT 344

Ctg654_12_NGS_assembly TTAGCTTCTTCTTTTAATAGGACGTTTGAACCGTTGTCCGACAGAAATGT 450

**************************************************

Ctg654_12_Sangerassembly CTGGAAATTCTGAATTACAGGCACATGTTGTCGGTTACAGAGGGCTTACC 394

Ctg654_12_NGS_assembly CTGGAAATTCTGAATTACAGGCACATGTTGTCGGTTACAGAGGGCTTACC 500

**************************************************

Ctg654_12_Sangerassembly AGGCAGACAGGGTCAGCCATACTGAGAATTCAAGTCACATACCACATTAA 444

Ctg654_12_NGS_assembly AGGCAGACAGGGTCAGCCATACTGAGAATTCAAGTCACATACCACATTAA 550

**************************************************

Ctg654_12_Sangerassembly CAGCCCTCCTCATTCAGACTGTCACTGTGACCTGTAGCATCTCTTTCAGC 494

Ctg654_12_NGS_assembly CAGCCCTCCTCATTCAGACTGTCACTGTGACCTGTAGCATCTCTTTCAGC 600

**************************************************

Ctg654_12_Sangerassembly TAGATGTAGCACTGAAACTGTGCTATAAGTGTAGTGTAACTGACTGCTTA 544

Ctg654_12_NGS_assembly TAGATGTAGCACTGAAACTGTGCTATAAGTGTAGTGTAACTGACTGCTTA 650

**************************************************

Ctg654_12_Sangerassembly CTTATTACTGTACATTATGTCTCATGTCTTTTTATAAGCAGGTACTGTGA 594

Ctg654_12_NGS_assembly CTTATTACTGTACATTATGTCTCATGTCTTTTTATAAGCAGGTACTGTGA 700

**************************************************

Ctg654_12_Sangerassembly TAGAAAAAGAAAAAGGTAGGTGGATGCTGCCATCAAAGAAACACTTGCAG 644

Ctg654_12_NGS_assembly TAGAAAAAGAAAAAGGTAGGTGGATGCTGCCATCAAAGAAACACTTGCAG 750

**************************************************

Ctg654_12_Sangerassembly GAAAATATTCTTACAAAAGTAATGTTTTGTTCTAAGAGATTATGTCCTTA 694

Ctg654_12_NGS_assembly GAAAATATTCTTACAAAAGTAATGTTTTGTTCTAAGAGATTATGTCCTTA 800

**************************************************

Ctg654_12_Sangerassembly TGTAATGTGAAACAAATTGAAGCCATGGAAAGGCGGGTAAACTTTTACTG 744

Ctg654_12_NGS_assembly TGTAATGTGAAACAAATTGAAGCCATGGAAAGGCGGGTAAACTTTTACTG 850

**************************************************

Ctg654_12_Sangerassembly TACAGTTAAAAAAATGGGTGAACTGAGATAAAGCTGGCTTATCACCATTC 794

Ctg654_12_NGS_assembly TACAGTTAAAAAAATGGGTGAACTGAGATAAAGCTGGCTTATCACCATTC 900

**************************************************

Ctg654_12_Sangerassembly ATGTATGTTATGTATGATAACTCTGCATTTCTCACAGGTAGTCAAGAAAG 844

Ctg654_12_NGS_assembly ATGTATGTTATGTATGATAACTCTGCATTTCTCACAGGTAGTCAAGAAAG 950

**************************************************

Ctg654_12_Sangerassembly TTGCAGTTAACCTCTGACCCTTGCCCTTCATCTAGCTTCTCATTTACCCC 894

Ctg654_12_NGS_assembly TTGCAGTTAACCTCTGACCCTTGCCCTTCATCTAGCTTCTCATTTACCCC 1000

**************************************************

Ctg654_12_Sangerassembly TGAAGCCAATAAGCAGTGATGCTGACCTGTTTACAGGCAATAAAGTAGTC 944

Ctg654_12_NGS_assembly TGAAGCCAATAAGCAGTGATGCTGACCTGTTTACAGGCAATAAAGTAGTC 1050

**************************************************

Ctg654_12_Sangerassembly AATTTTCAAACTTAAACATGACATCATTGGTCAAAGATACATCATGTGAG 994

Ctg654_12_NGS_assembly AATTTTCAAACTTAAACATGACATCATTGGTCAAAGATACATCATGTGAG 1100

**************************************************

Ctg654_12_Sangerassembly TGGTAACACAACAAGTGGTCACACTTCTGAGCCCTTATCAAACTTGTGCT 1044

Ctg654_12_NGS_assembly TGGTAACACAACAAGTGGTCACACTTCTGAGCCCTTATCAAACTTGTGCT 1150

**************************************************

Ctg654_12_Sangerassembly CTTATTTCTGTTCACTTCCCCATATCTCAGGACATTTGTTCATTGTTTCT 1094

Ctg654_12_NGS_assembly CTTATTTCTGTTCACTTCCCCATATCTCAGGACATTTGTTCATTGTTTCT 1200

**************************************************

Ctg654_12_Sangerassembly CATGTCACTTAATCTGGAAAACACTGAGACAAATGCGAGCAAATTACTTT 1144

Ctg654_12_NGS_assembly CATGTCACTTAATCTGGAAAACACTGAGACAAATGCGAGCAAATTACTTT 1250

**************************************************

Ctg654_12_Sangerassembly GAGACCGAAAGCAAAGAAGACCACTGAATGGTCATTCAGTGCAACTTATT 1194

Ctg654_12_NGS_assembly GAGACCGAAAGCAAAGAAGACCACTGAATGGTCATTCAGTGCAACTTATT 1300

**************************************************

Ctg654_12_Sangerassembly TGATGGACTTTCCCTCTGACACAATTATTAGTGTGGGCACAGGGTTGTTT 1244

Ctg654_12_NGS_assembly TGATGGACTTTCCCTCTGACACAATTATTAGTGTGGGCACAGGGTTGTTT 1350

**************************************************

Ctg654_12_Sangerassembly TTAGCACATTGTGGCCTGAATGGTGGAACTTCATCTTCTCTAATTGTCAC 1294

Ctg654_12_NGS_assembly TTAGCACATTGTGGCCTGAATGGTGGAACTTCATCTTCTCTAATTGTCAC 1400

**************************************************

Ctg654_12_Sangerassembly ACTTACTGCAATTCATGAAAAATACAAACCTAATGCAAAACATGTACAGT 1344

Ctg654_12_NGS_assembly ACTTACTGCAATTCATGAAAAATACAAACCTAATGCAAAACATGTACAGT 1450

**************************************************

Ctg654_12_Sangerassembly ATTTTCAGTAAACATGGTATTTTTTCCTCTGCTCCTCCTTTGGCTGGAAA 1394

Ctg654_12_NGS_assembly ATTTTCAGTAAACATGGTATTTTTTCCTCTGCTCCTCCTTTGGCTGGAAA 1500

**************************************************

Ctg654_12_Sangerassembly GTGGCCAACTGATAATGCAGACTTCTCTGGTCTGAGCTGCAGCTTAACCC 1444

Ctg654_12_NGS_assembly GTGGCCAACTGATAATGCAGACTTCTCTGGTCTGAGCTGCAGCTTAACCC 1550

**************************************************

Ctg654_12_Sangerassembly CAGTCTTTCTGGAAATCCTTTCTGCTCAGACCACTTGCTGACTTCAAGCT 1494

Ctg654_12_NGS_assembly CAGTCTTTCTGGAAATCCTTTCTGCTCAGACCACTTGCTGACTTCAAGCT 1600

**************************************************

Ctg654_12_Sangerassembly ATAGATGATGATCCAATCATTTTATCTAATGAAGATTTATGTTTTCTAGA 1544

Ctg654_12_NGS_assembly ATAGATGATGATCCAATCATTTTATCTAATGAAGATTTATGTTTTCTAGA 1650

**************************************************

Ctg654_12_Sangerassembly ATGTCTGACACCCCGTTCCGAAAGTGCACTATACATTGTTGCTGCCTTGT 1594

Ctg654_12_NGS_assembly ATGTCTGACACCCCGTTCCGAAAGTGCACTATACATTGTTGCTGCCTTGT 1700

**************************************************

Ctg654_12_Sangerassembly GTCATGGGCTATTTCTCCACTGCTGGAGAAACTGACACCTCTGTCTGTCT 1644

Ctg654_12_NGS_assembly GTCATGGGCTATTTCTCCACTGCTGGAGAAACTGACACCTCTGTCTGTCT 1750

**************************************************

Ctg654_12_Sangerassembly GATTGTTGGATGTCAGGGTTAGAAGTCCTGACTAACAACAAAACCATCAT 1694

Ctg654_12_NGS_assembly GATTGTTGGATGTCAGGGTTAGAAGTCCTGACTAACAACAAAACCATCAT 1800

**************************************************

Ctg654_12_Sangerassembly CAGTCTTATAGTGGTTCGCTGGTATCCTGGCTTTCTTAATCACATTTGCT 1744

Ctg654_12_NGS_assembly CAGTCTTATAGTGGTTCGCTGGTATCCTGGCTTTCTTAATCACATTTGCT 1850

**************************************************

Ctg654_12_Sangerassembly GTTAAGTTTGACCTCTGATAACAGGCTGAATTTGGACAGATGTTTCTTTT 1794

Ctg654_12_NGS_assembly GTTAAGTTTGACCTCTGATAACAGGCTGAATTTGGACAGATGTTTCTTTT 1900

**************************************************

Ctg654_12_Sangerassembly TATAATCGGCATTTGCCAGATCGGCTTTTCGCTCTATAGTGCAGCTATAT 1844

Ctg654_12_NGS_assembly TATAATCGGCATTTGCCAGATCGGCTTTTCGCTCTATAGTGCAGCTATAT 1950

**************************************************

Ctg654_12_Sangerassembly CCCTCCCAGCTCTCAAAAGGACACAAAAACTAGACCCACTACCATGTTTG 1894

Ctg654_12_NGS_assembly CCCTCCCAGCTCTCAAAAGGACACAAAAACTAGACCCACTACCATGTTTG 2000

**************************************************

Ctg654_12_Sangerassembly GAGTGCTTTTCACTGAAGGTTAAGGGCATGCTATTCTCAAGCGGCTCGTT 1944

Ctg654_12_NGS_assembly GAGTGCTTTTCACTGAAGGTTAAGGGCATGCTATTCTCAAGCGGCTCGTT 2050

**************************************************

Ctg654_12_Sangerassembly AATCCCAGCATTTGTTTTCTTTTTTCCTCTCAGTTCTGATGCTGTCAGTG 1994

Ctg654_12_NGS_assembly AATCCCAGCATTTGTTTTCTTTTTTCCTCTCAGTTCTGATGCTGTCAGTG 2100

**************************************************

Ctg654_12_Sangerassembly TTAGTGGTTTTAAATGCATTGTTTAGGCCTGTTTTTTTTTTTAAACTGCT 2044

Ctg654_12_NGS_assembly TTAGTGGTTTTAAATGCATTGTTTAGGCCTGTTTTTTTTTTTAAACTGCT 2150

**************************************************

Ctg654_12_Sangerassembly GTTAAAGATCCCAAAATGGGACATGGACCCTCTCCATGGGGCAGGGTGGT 2094

Ctg654_12_NGS_assembly GTTAAAGATCCCAAAATGGGACATGGACCCTCTCCATGGGGCAGGGTGGT 2200

**************************************************

Ctg654_12_Sangerassembly CAGTGTTTTGGCAATCTTGTGTCTGCTCAAAAAGTTAAACTAGCAAAGCT 2144

Ctg654_12_NGS_assembly CAGTGTTTTGGCAATCTTGTGTCTGCTCAAAAAGTTAAACTAGCAAAGCT 2250

**************************************************

Ctg654_12_Sangerassembly GTGGTTATGTATTTGGCTCTACATGCATCTGTCTTAGACAATTAAGGAAA 2194

Ctg654_12_NGS_assembly GTGGTTGTGTATTTGGCTCTACATGCATCTGTCTTAGACAATTAAGGAAA 2300

****** *******************************************

Ctg654_12_Sangerassembly GGAGATTGACCAGTGGGCCCTGTGAGCAGTAATTAGTTATTACTATGATG 2244

Ctg654_12_NGS_assembly GGAGATTGACCAGTGGGCCCTGTGAGCAGTAATTAGTTATTACTATGATG 2350

**************************************************

Ctg654_12_Sangerassembly AATAATGAGCACAATCAACAAGCTACACAAATGATGTTGTGCTTCTTTTT 2294

Ctg654_12_NGS_assembly AATAATGAGCACAATCAACAAGCTACACAAATGATGTTGTGCTTCTTTTT 2400

**************************************************

Ctg654_12_Sangerassembly TCTCTTGCTCTCTGCTGCTTCCTCCATAATGCAGCAGAGATGCCATAAAA 2344

Ctg654_12_NGS_assembly TCTCTTGCTCTCTGCTGCTTCCTCCATAATGCAGCAGAGATGCCATAAAA 2450

**************************************************

Ctg654_12_Sangerassembly GTAGCTGAGAGGAAAAAGGAAAGCTGCACCCCCAGGCTATATCATTATAT 2394

Ctg654_12_NGS_assembly GTAGCTGAGAGGAAAAAGGAAAGCTGCACCCCCAGGCTATATCATTATAT 2500

**************************************************

Ctg654_12_Sangerassembly CACAGCTCATATGAGCCCAGCTGCTCCAAACCACCGTCTTACCTGCGTTT 2444

Ctg654_12_NGS_assembly CACAGCTCATATGAGCCCAGCTGCTCCAAACCACCGTCTTACCTGCGTTT 2550

**************************************************

Ctg654_12_Sangerassembly CTCCATCCGTCCCCCTGTGAATGTTGTTAATAACATTTGGGCAAATGTAC 2494

Ctg654_12_NGS_assembly CTCCATCCGTCCCCCTGTGAATGTTGTTAATAACATTTGGGCAAATGTAC 2600

**************************************************

Ctg654_12_Sangerassembly CTACACAACATCCTCCCTGATTTAAAAATAGCAGGGGATATGATTGATTG 2544

Ctg654_12_NGS_assembly CTACACAACATCCTCCCTGATTTAAAAATAGCAGGGGATATGATTGATTG 2650

**************************************************

Ctg654_12_Sangerassembly TGAGTTGCATCACTAACACTATCAAAGCCTTTGTTGCTAAGGGGATCAGA 2594

Ctg654_12_NGS_assembly TGAGTTGCATCACTAACACTATCAAAGCCTTTGTTGCTAAGGGGATCAGA 2700

**************************************************

Ctg654_12_Sangerassembly GTGTGTGACTCAGGATAACACTGTTCATTGTGACTCAGAAAGCGGCTGTC 2644

Ctg654_12_NGS_assembly GTGTGTGACTCAGGATAACACTGTTCATTGTGACTCAGAGAGCGGCTGTC 2750

*************************************** **********

Ctg654_12_Sangerassembly TCTGTTTTGGGATGTATGGGGGTGTGGAGGAATGAGATGTGCTGTGCAGT 2694

Ctg654_12_NGS_assembly TCTGTTTTGGGATGTATGGGGGTGTGGAGGAATGAGATGTGCTGTGCAGT 2800

**************************************************

Ctg654_12_Sangerassembly CTTGTCCACAGCCTTTTCATTTCTGCTTTCCAGTTATCTCAGTAAAGGCA 2744

Ctg654_12_NGS_assembly CTTGTCCACAGCCTTTTCATTTCTGCTTTCCAGTTATCTCAGTAAAGGCA 2850

**************************************************

Ctg654_12_Sangerassembly GAACATGCCAGAGTTTCTGTCTGGTGTCTAGAGCACATGAACAAGGGAAA 2794

Ctg654_12_NGS_assembly GAACATGCCAGAGTTTCTGTCTGGTGTCTAGAGCACATGAACAAGGGAAA 2900

**************************************************

Ctg654_12_Sangerassembly ACCTATGAGCTTTATCCTGTTTATTAGAATCGGTGCTGATTTTTTTTTTT 2844

Ctg654_12_NGS_assembly ACCTATGAGCTTTATCCTGTTTATTAGAATCGGTGCAGATTTTTTTTTTT 2950

************************************ *************

Ctg654_12_Sangerassembly TTTC-AGTTTTCAGCACTGCAGTACCCCCCTCAATGCTGTGGACGTGTTT 2893

Ctg654_12_NGS_assembly TTTCCAGTTTTCAGCACTGCAGTACCCCCCTCAATGCTGTGGACGTGTTT 3000

**** *********************************************

Ctg654_12_Sangerassembly TGCAAATATTGTTTGTTTGCGTATAGTGATGTCATTGTTTGATCAGGTAA 2943

Ctg654_12_NGS_assembly TGCAAATATTGTTTGTTTGCGTATAGTGATGTCATTGTTTGATCAGGTAA 3050

**************************************************

Ctg654_12_Sangerassembly TAGCGTTTAATCGAGTTGACAGGTTGAGATGAAGTGACTGCAGACAGTGC 2993

Ctg654_12_NGS_assembly TAGCGTTTAATCGAGTTGACAGGTTGAGATGAAGTGACTGCAGACAGTGC 3100

**************************************************

Ctg654_12_Sangerassembly TCTGTTCACTGGAAATCGGTTTTATGTCCTTGCCAGTTACACAGAGTAAA 3043

Ctg654_12_NGS_assembly TCTGTTCACTGGAAATCGGTTTTATGTCCTTGCCAGTTACACAGAGTAAA 3150

**************************************************

Ctg654_12_Sangerassembly TCTAATTAAATGCGTAACATGTGCTTCAGAGAGAGACATATCCTTCTCGT 3093

Ctg654_12_NGS_assembly TCTAATTAAATGCGTAACATGTGCTTCAGAGAGAGACATATCCTTCTCGT 3200

**************************************************

Ctg654_12_Sangerassembly GACCTGTTGTCTTTTTTTTCCCCTCTTCCTCTATTCAAAAATAAACCTTT 3143

Ctg654_12_NGS_assembly GACCTGTTGTCTTTTTTTTCCCCTCTTCCTCTATTCAAAAATAAACCTTT 3250

**************************************************

Ctg654_12_Sangerassembly TGAGAGTTACATAACTATTCACACACGGTTTCTGTCTTTTAAGGGTCAAA 3193

Ctg654_12_NGS_assembly TGAGAGTTACATAACTATTCACACACGGTTTCTGTCTTTTAAGGGTCAAA 3300

**************************************************

Ctg654_12_Sangerassembly TGCTGTGTTGTTTCTATCTCTGCTCATGTGATACCCTTTTCAGGCACTGT 3243

Ctg654_12_NGS_assembly TGCTGTGTTGTTTCTATCTCTGCTCATGTGATACCCTTTTCAGGCACTGT 3350

**************************************************

Ctg654_12_Sangerassembly TTACTAACCATTCAAGATAGATCTAATTCATTCATTCTTGTCTCATGTTA 3293

Ctg654_12_NGS_assembly TTACTAACCATTCAAGATAGATCTAATTCATTCATTCTTGTCTCATGTTA 3400

**************************************************

Ctg654_12_Sangerassembly GTCAAAGCATCTTCAGGTCATCCTCAAACATAAATATCAGTTCTGAAGTA 3343

Ctg654_12_NGS_assembly GTCAAAGCATCTTCAGGTCATCCTCAAACATAAATATCTGTTCTGAAGTA 3450

************************************** ***********

Ctg654_12_Sangerassembly TGACAGAATGATGTAATTATTTGATCATGTGGAGATAGTGATGCTGAGGT 3393

Ctg654_12_NGS_assembly TGACAGAATGATGTAATTATTTGATCATGTGGAGATAGTGATGCTGAGGT 3500

**************************************************

Ctg654_12_Sangerassembly TTTTAGTATGACCTTTATCTATGCTCAGAGAAGTTTAATGCAGCTTGTCA 3443

Ctg654_12_NGS_assembly TTTTAGTATGACCTTTATCTATGCTCAGAGAAGTTTAATGCAGCTTGTCA 3550

**************************************************

Ctg654_12_Sangerassembly GGATGCCAAATAAAACTTCAAACGTCAGAAGGGCGGTTTGTTTTCCTGCA 3493

Ctg654_12_NGS_assembly GGATGCCAAATAAAACTTCAAACGTCAGAAGGGCGGTTTGTTTTCCTGCA 3600

**************************************************

Ctg654_12_Sangerassembly ACAGCTGATATACTGAAAACACAGAAAGCCCAGGATAGTAATAGTAATAT 3543

Ctg654_12_NGS_assembly ACAGCTGATATACTGAAAACACAGAAAGCCCAGGATAGTAATAGTAATAT 3650

**************************************************

Ctg654_12_Sangerassembly CATGAATTCAGCAAAAATGGTCGTCAAAAAAAGCATGTAGTAATACAGTG 3593

Ctg654_12_NGS_assembly CATGAATTCAGCAAAAATGGTCGTCAAAAAAAGCATGTAGTAATACAGTG 3700

**************************************************

Ctg654_12_Sangerassembly ACCCTCATGAGCACACGTGAGTCAAAATGTCTGATGCTGATGGAAGGTTG 3643

Ctg654_12_NGS_assembly ACCCTCATGAGCACACGTGAGTCAAAATGTCTGATGCTGATGGAAGGTTG 3750

**************************************************

Ctg654_12_Sangerassembly TTAAAGGCATCTCAGCAAAATTCACAAACCCATAGTAGAAAAGCTGACAA 3693

Ctg654_12_NGS_assembly TTAAAGGCATCTCAGCAAAATTCACAAACCCATAGTAGAAAAGCTGACAA 3800

**************************************************

Ctg654_12_Sangerassembly CACACTCACAATCTGCCATCCTTTAGCTGCTTGTGGTGCTGATCTCCACA 3743

Ctg654_12_NGS_assembly CACACTCACAATCTGCCATCCTTTAGCTGCTTGTGGTGCTGATCTCCACA 3850

**************************************************

Ctg654_12_Sangerassembly CTGATTATAGTGCTGTTGTCAGGAAGATGCTTAACGTTTAACTACCATAA 3793

Ctg654_12_NGS_assembly CTGATTATAGTGCTGTTGTCAGGAAGATGCTTAACGTTTAACTACCATAA 3900

**************************************************

Ctg654_12_Sangerassembly ACATCTTGGAGTGAGGTCATCCTTTGTGGTCGTAGGTTTTGCCTAGTCGA 3843

Ctg654_12_NGS_assembly ACATCTTGGAGTGAGGTCATCCTTTGTGGTCGTAGGTTTTGCCTAGTCGA 3950

**************************************************

Ctg654_12_Sangerassembly CAGGCTTAATTCGCCCCTAAGTTGTCTCCGGGAGAACCTTTGGCCGCGTT 3893

Ctg654_12_NGS_assembly CAGGCTTAATTCGCCCCTAAGTTGTCTCCGGGAGAACCTTTGGCCGCGTT 4000

**************************************************

Ctg654_12_Sangerassembly TAACTCACTTTAATGGGATTATATTGCGGCAGCTGGGGGAAAATGCTGAC 3943

Ctg654_12_NGS_assembly TAACTCACTTTAATGGGATTATATTGCGGCAGCTGGGGGAAAATGCTGAC 4050

**************************************************

Ctg654_12_Sangerassembly TTCAGTTCTCAGCACTCGACTAAGCAGTTTGTTGACTGTGGCGGCGTCTG 3993

Ctg654_12_NGS_assembly TTCAGTTCTCAGCACTCGACTAAGCAGTTTGTTGACTGTGGCGGCGTCTG 4100

**************************************************

Ctg654_12_Sangerassembly ATGTTTTGGGCATAGCTGTAATCTGGGCCGAGGTTCCCTCCCCTGCTACA 4043

Ctg654_12_NGS_assembly ATGTTTTGGGCATAGCTGTAATCTGGGCCGAGGTTCCCTCCCCTGCTACA 4150

**************************************************

Ctg654_12_Sangerassembly GAGTACATTGGGGCGAGGCGCTTACGTAACCTCCGCCGTCATCTTTTTCA 4093

Ctg654_12_NGS_assembly GAGTACATTGGGGCGAGGCGCTTACGTAACCTCCGCCGTCATCTTTTTCA 4200

**************************************************

Ctg654_12_Sangerassembly TTCACTGCTGGACTGGCATCCACGGTATGAGCCATGACCTGCCCCGCACA 4143

Ctg654_12_NGS_assembly TTCACTGCTGGACTGGCATCCACGGTATGAGCCATGACCTGCCCCGCACA 4250

**************************************************

Ctg654_12_Sangerassembly CTAGGGTTTTGAAGTTAGCGCTAAATCCAGGCTTAAGTTGTTAGTTATGA 4193

Ctg654_12_NGS_assembly CTAGGGTTTTGAAGTTAGCGCTAAATCCAGGCTTAAGTTGTTAGTTATGA 4300

**************************************************

Ctg654_12_Sangerassembly AGAAGTTTCACTGATGCCAGAAGTAGCAGGTCACATTGCATTAGTCTCAA 4243

Ctg654_12_NGS_assembly AGAAGTTTCACTGATGCCAGAAGTAGCAGGTCACATTGCATTAGTCTCAA 4350

**************************************************

Ctg654_12_Sangerassembly TTGCCTCCAGTTGTATTAAATTTTTGATCTGAAAGTGCTTAATCCCGATT 4293

Ctg654_12_NGS_assembly TTGCCTCCAGTTGTATTAAATTTTTGATCTGAAAGTGCTTAATCCCGATT 4400

**************************************************

Ctg654_12_Sangerassembly TATGTTTAGTTTTCAGTGGGATTGTTTTGACATGACATGATCATAAAATG 4343

Ctg654_12_NGS_assembly TATGTTTAGTTTTCAGTGGGATTGTTTTGACATGACATGATCATAAAATG 4450

**************************************************

Ctg654_12_Sangerassembly AAATTGCGTAAAGGCTATTTATGTGATTTTTTTTCCAAAATAAAGTGCAG 4393

Ctg654_12_NGS_assembly AAATTGCGTAAAGGCTATTTATGTGATTTTTTTTCCAAAATAAAGTGCAG 4500

**************************************************

Ctg654_12_Sangerassembly GTGATCTGGGACCACTGACTACAACAACAACAAGGTTTATTTACAACTAT 4443

Ctg654_12_NGS_assembly GTGATCTGGGACCACTGACTACAACAACAACAAGGTTTATTTACAACTAT 4550

**************************************************

Ctg654_12_Sangerassembly GTCAACTACAAAAGATGACAAGTCTCTCGAGTAGGCAGGGAAATAAAAGA 4493

Ctg654_12_NGS_assembly GTCAACTACAAAAGATGACAAGTCTCTCGAGTAGGCAGGGAAATAAAAGA 4600

**************************************************

Ctg654_12_Sangerassembly ATAAAAACTGGATAGAAATAAAATCATCAGCTAAGTGCAGCATATGGCGA 4543

Ctg654_12_NGS_assembly ATAAAAACTGGATAGAAATAAAATCATCAGCTAAGTGCAGCATATGGCGA 4650

**************************************************

Ctg654_12_Sangerassembly GAAAAACCTGGATGTGTTGTCAGTGTGCTGGCCAAAGTGCACATTAAACA 4593

Ctg654_12_NGS_assembly GAAAAACCTGGATGTGTTGTCAGTGTGCTGGCCAAAGTGCACATTAAACA 4700

**************************************************

Ctg654_12_Sangerassembly GGTTATTTATCAGTTGAGCCAGTTATGGACACAGAGGTGATCACTGATGC 4643

Ctg654_12_NGS_assembly GGTTATTTATCAGTTGAGCCAGTTATGGACACAGAGGTGATCACTGATGC 4750

**************************************************

Ctg654_12_Sangerassembly GTCATTGAAGCATATTACTCATACAGTGTAAGGTTACGGGGCTGTAGGCC 4693

Ctg654_12_NGS_assembly GTCATTGAAGCATATTACTCATACAGTGTAAGGTTACGGGGCTGTAGGCC 4800

**************************************************

Ctg654_12_Sangerassembly ATATACTTCGTCATTAGCTGGCAAAGCAACTTTTAGAGGGTGTCCTAATG 4743

Ctg654_12_NGS_assembly ATATACTTCGTCATTAGCTGGCAAAGCAACTTTTAGAGGGTGTCCTAATG 4850

**************************************************

Ctg654_12_Sangerassembly AATAAGGGGTGGAGACATAGCACTTAAGTATATTGTCTTTCATCGGGTAG 4793

Ctg654_12_NGS_assembly AATAAGGGGTGGAGACATAGCACTTAAGTATATTGTCTTTCATCGGGTAG 4900

**************************************************

Ctg654_12_Sangerassembly CTCTGCCTTTGTTTAAACCCAGTGTCTGGTATGTTTTTCCAGGGAGGTCA 4843

Ctg654_12_NGS_assembly CTCTGCCTTTGTTTAAACCCAGTGTCTGGTATGTTTTTCCAGGGAGGTCA 4950

**************************************************

Ctg654_12_Sangerassembly CTGATATGAATTTAAAATAGATATAGAGGAGTCTTTATGTTTCTGCTTAA 4893

Ctg654_12_NGS_assembly CTGATATGAATTTAAAATAGATATAGAGGAGTCTTTATGTTTCTGCTTAA 5000

**************************************************

Ctg654_12_Sangerassembly AGTAATATAGATTTGTTTGAAACAGGAGGATTGGTTTGATCAATGATAGT 4943

Ctg654_12_NGS_assembly AGTAATATAGATTTGTTTGAAACAGGAGGATTGGCTTGATCAATGATAGT 5050

********************************** ***************

Ctg654_12_Sangerassembly GTTCATGGCAGCAAAAACAGCAAGATGCTGACAAAAATCTTGCTCTTTTT 4993

Ctg654_12_NGS_assembly GTTCATGGCAGCAAAAACAGCAAGATGCTGACAAAAATCTTGCTCTTTTT 5100

**************************************************

Ctg654_12_Sangerassembly TAACCTTTCTTTCTATTTGCCACATGTATTTGCCTGTTGATGCTTATTTT 5043

Ctg654_12_NGS_assembly TAACCTTTCTTTCTATTTGCCACATGTATTTGCCTGTTGATGCTTATTTT 5150

**************************************************

Ctg654_12_Sangerassembly AGTTCTCCCTAAATTATACTCATTGTGGGAAGTTGAAGGTTTAGAACAGT 5093

Ctg654_12_NGS_assembly AGTTCTCCCTAAATTATACTCATTGTGGGAAGTTGAAGGTTTAGAACAGT 5200

**************************************************

Ctg654_12_Sangerassembly GAATTTTACGGATCACAGACAGACTGACCTGGCCCGCTTAAAATGCATAT 5143

Ctg654_12_NGS_assembly GAATTTTACGGATCACAGACAGACTGACCTGGCCCGCTTAAAATGCATAT 5250

**************************************************

Ctg654_12_Sangerassembly TTGTTTATTTCCCCTCATCTCACTCAAAGTAGTCAGGAAGGGAGGAGGGG 5193

Ctg654_12_NGS_assembly TTGTTTATTTCCCCTCATCTCACTCAAAGTAGTCAGGAAGGGAGGAGGGG 5300

**************************************************

Ctg654_12_Sangerassembly GGCGGATGAAAAATATATTGCTGTTCCACTTTAACGCAGGCATGTAGAAT 5243

Ctg654_12_NGS_assembly GGCGGATGAAAAATATATTGCTGTTCCACTTTAACGCAGGCATGTAGAAT 5350

**************************************************

Ctg654_12_Sangerassembly ATTCTTTCTGGCTTAGCGACTCAATTTCTTTAGAGTTCGAAGCTGTGGTC 5293

Ctg654_12_NGS_assembly ATTCTTTCTGGCTTAGCGACTCAATTTCTTTAGAGTTCGAAGCTGTGGTC 5400

**************************************************

Ctg654_12_Sangerassembly AATCTGTCATCGGGAGGCCAGTCGCTGAAAGGCCTCCTGCCACATGGTAG 5343

Ctg654_12_NGS_assembly AATCTGTCATCGGGAGGCCAGTCGCTGAAAGGCCTCCTGCCACATGGTAG 5450

**************************************************

Ctg654_12_Sangerassembly TATGGGCACGTGGAGGGTGTGTGTGTGTGTGTGTAATATAGGTCAGCATT 5393

Ctg654_12_NGS_assembly TATGGGCACGTGGAGGGTGTGTGTGTGTGTGTGTAATATAGGTCAGCATT 5500

**************************************************

Ctg654_12_Sangerassembly GTGCAGCACTGCAGTCCTGTCCTGTGGGCCTGTCTCTCTCTTTGTCCCAT 5443

Ctg654_12_NGS_assembly GTGCAGCACTGCAGTCCTGTCCTGTGGGCCTGTCTCTCTCTTTGTCCCAT 5550

**************************************************

Ctg654_12_Sangerassembly CTGCTGATGTGACGGGCCAACTTCAGCGCTCATGTCAATGTGATTTCTCT 5493

Ctg654_12_NGS_assembly CTGCTGATGTGACGGGCCAACTTCAGCGCTCATGTCAATGTGATTTCTCT 5600

**************************************************

Ctg654_12_Sangerassembly CTTTCTCTCACCCTGGTTACATTTTTTTCCTAAGGGATCAGTGGGAAACG 5543

Ctg654_12_NGS_assembly CTTTCTCTCACCCTGGTTACATTTTTTTCCTAAGGGATCAGTGGGAAACG 5650

**************************************************

Ctg654_12_Sangerassembly ATGGGGGATATTCCACCAATTAAAACAGCTGGCATTTAGATGGAAGGAAG 5593

Ctg654_12_NGS_assembly ATGGGGGATATTCCACCAATTAAAACAGCTGGCATTTAGATGGAAGGAAG 5700

**************************************************

Ctg654_12_Sangerassembly CATGCGCTCGCCCAAACTAAATCACCAGCTGCTTTCAGAGAGTCTTATCA 5643

Ctg654_12_NGS_assembly CATGCGCTCGCCCAAACTAAATCACCAGCTGCTTTCAGAGAGTCTTATCA 5750

**************************************************

Ctg654_12_Sangerassembly TGTGATAATAATCTTATGAAACGCACAGAACACAACAACCCCCGCTTTCA 5693

Ctg654_12_NGS_assembly TGTGATAATAATCTTATGAAACGCACAGAACACAACAACCCCCGCTTTCA 5800

**************************************************

Ctg654_12_Sangerassembly AGCCTACTGCGGATGGACAGTGACCTTTTCAGCAGAATCGCATCTCAGTA 5743

Ctg654_12_NGS_assembly AGCCTACTGCGGATGGACAGTGACCTTTTCAGCAGAATCGCATCTCAGTA 5850

**************************************************

Ctg654_12_Sangerassembly AACACTTGAAAATGGCTGGAGCAAAAAAAGTCGGTGTGATTCTTGACCCC 5793

Ctg654_12_NGS_assembly AACACTTGAAAATGGCTGGAGCAAAAAAAGTCGGTGTGATTCTTGACCCC 5900

**************************************************

Ctg654_12_Sangerassembly CCCTCCTCCCGTTGTCCCCATTTCCCTGTCTCACTTTGTTGCTGTTTTTT 5843

Ctg654_12_NGS_assembly CCCTCCTCCCGTTGTCCCCATTTCCCTGTCTCACTTTGTTGCTGTTTTTT 5950

**************************************************

Ctg654_12_Sangerassembly GCTCGCATGCAGCTCAGCACGTCTAGTGGTCATTCAGCTCAGGGCATTTC 5893

Ctg654_12_NGS_assembly GCTCGCATGCAGCTCAGCACGTCTAGTGGTCATTCAGCTCAGGGCATTTC 6000

**************************************************

Ctg654_12_Sangerassembly TCTCTCTTTGCCTGAGCTTTGTCTGTTCCCAAGCTTCTTAAAAGAGCTCA 5943

Ctg654_12_NGS_assembly TCTCTCTTTGCCTGAGCTTTGTCTGTTCCCAAGCTTCTTAAAAGAGCTCA 6050

**************************************************

Ctg654_12_Sangerassembly TCCAGCTGACCGACCCCCTTAGTCAGACCAGCCTGCCAAGCAAGGCTGAT 5993

Ctg654_12_NGS_assembly TCCAGCTGACCGACCCCCTTAGTCAGACCAGCCTGCCAAGCAAGGCTGAT 6100

**************************************************

Ctg654_12_Sangerassembly TCCAGGAATAGGACTTCTTGTGGGTCTCCAGATTTAGTATTAGTACTTTT 6043

Ctg654_12_NGS_assembly TCCAGGAATAGGACTTCTTGTGGGTCTCCAGATTTAGTATTAGTACTTTT 6150

**************************************************

Ctg654_12_Sangerassembly TAAAGCTTTCCCCTATTTTTAGTTTTGTTTTAGTTCTGCACTGGGAGGAG 6093

Ctg654_12_NGS_assembly TAAAGCTTTCCCCTATTTTTAGTTTTGTTTTAGTTCTGCACTGGGAGGAG 6200

**************************************************

Ctg654_12_Sangerassembly GGTGCGTTATCATTAGGCACCTGCTTATTAGAGATTATGAATATATAAAT 6143

Ctg654_12_NGS_assembly GGTGCGTTATCATTAGGCACCTGCTTATTAGAGATTATGAATATATAAAT 6250

**************************************************

Ctg654_12_Sangerassembly TATAATTGCACGCTTTTTGAAATGGAGCCATATAGTTAAAAGTCCCATAA 6193

Ctg654_12_NGS_assembly TATAATTGCACGCTTTTTGAAATGGAGCCATATAGTTAAAAGTCCCATAA 6300

**************************************************

Ctg654_12_Sangerassembly GCTTACAACTCTGTACGTAAAATAAGAGGAATCTGTCACTACTCAAACAA 6243

Ctg654_12_NGS_assembly GCTTACAACTCTGTACGTAAAATAAGAGGAATCTGTCACTACTCAAACAA 6350

**************************************************

Ctg654_12_Sangerassembly TTGAAACCAAATACTGCCTAGTAACTTTTTTTGTCCTGCATCAGGTCAGC 6293

Ctg654_12_NGS_assembly TTGAAACCAAATACTGCCTAGTAACTTTTTTTGTCCTGCATCAGGTCAGC 6400

**************************************************

Ctg654_12_Sangerassembly TGACCAACACAAGAAAATCTTCTGCATGTTTTATTTTGAGTGTTTCATCA 6343

Ctg654_12_NGS_assembly TGACCAACACAAGAAAATCTTCTGCATGTTTTATTTTGAGTGTTTCATCA 6450

**************************************************

Ctg654_12_Sangerassembly GTGTGTGACTGTCTCTCATTTGCACTGAATCAGCAGACAACAGTAACCAA 6393

Ctg654_12_NGS_assembly GTGTGTGACTGTCTCTCATTTGCACTGAATCAGCAGACAACAGTAACCAA 6500

**************************************************

Ctg654_12_Sangerassembly GTGTTTAGCTGTAGGCCACATACTGAGTCATACAGTGCCTTTTTTTTTTT 6443

Ctg654_12_NGS_assembly GTGTTTAGCTGTAGGCCACATACTGAGTCATACAGTGCCTTTTTTTTTTT 6550

**************************************************

Ctg654_12_Sangerassembly TTTTTTTTTAATGTTCAGATTTACTTTTTGGTGTAGTGGCTGACTATGCA 6493

Ctg654_12_NGS_assembly TTTTTTTTTAATGTTCAGATTTACTTTTTGGTGTAGTGGCTGACTATGCA 6600

**************************************************

Ctg654_12_Sangerassembly GTCACAGCCCTCCTAGATATAGCTGAGTCCTCTTGAAGTAATAATCAGAG 6543

Ctg654_12_NGS_assembly GTCACAGCCCTCCTAGATATAGCTGAGTCCTCTTGAAGTAATAATCAGAG 6650

**************************************************

Ctg654_12_Sangerassembly TAAACCAAGCATTTTATCAAAAGAAATAAATAAATTAAACGCCAGCCACT 6593

Ctg654_12_NGS_assembly TAAACCAAGCATTTTATCAAAAGAAATAAATAAATTAAACGCCAGCCACT 6700

**************************************************

Ctg654_12_Sangerassembly GTTCTCCACTCTGTCTTCGGCTTTTGGAACACCAGACTAATTGATTTTTA 6643

Ctg654_12_NGS_assembly GTTCTCCACTCTGTCTTCGGCTTTTGGAACACCAGACTAATTGATTTTTA 6750

**************************************************

Ctg654_12_Sangerassembly GGAAATGTTGCAGTGCAACCAGAGCTGTCTTTCCAAGTGCTGCAAGGCAG 6693

Ctg654_12_NGS_assembly GGAAATGTTGCAGTGCAACCAGAGCTGTCTTTCCAAGTGCTGCAAGGCAG 6800

**************************************************

Ctg654_12_Sangerassembly GCTTACAGGAAACTAGTGACGCGCGCACACACACACATGGAGCTTACACA 6743

Ctg654_12_NGS_assembly GCTTACAGGAAACTAGTGACGCGCGCACACACACACATGGAGCTTACACA 6850

**************************************************

Ctg654_12_Sangerassembly CTCATATAGGGTAGGGGCTTATTTCCTTGGAGAGATTATGGTAGCGATGT 6793

Ctg654_12_NGS_assembly CTCATATAGGGTAGGGGCTTATTTCCTTGGAGAGATTATGGTAGCGATGT 6900

**************************************************

Ctg654_12_Sangerassembly TGGGGGTTATGTTGTAATCGCTGTGTTGCATCTTAATGTTAGGCCCCCAC 6843

Ctg654_12_NGS_assembly TGGGGGTTATGTTGTAATCGCTGTGTTGCATCTTAATGTTAGGCCCCCAC 6950

**************************************************

Ctg654_12_Sangerassembly TGGAGGCTGATGTGGAAATTGTGCAGTGAGGTGGGCAGGAAATTGTGCGT 6893

Ctg654_12_NGS_assembly TGGAGGCTGATGTGGAAATTGTGCAGAGAGGTGGGCAGGAAATTGTGCGT 7000

************************** ***********************

Ctg654_12_Sangerassembly TCCTAAAATAAAGATGGAGAACAAAGAATGAGATAGAAAATTACAGTGTG 6943

Ctg654_12_NGS_assembly TCCTAAAATAAAGATGGAGAACAAAGAATGAGATAGAAAATTACAGTGTG 7050

**************************************************

Ctg654_12_Sangerassembly TGGAGCATGTGATTATGGAGCAGGATCACATGGATTTTCCCGGCATTTGA 6993

Ctg654_12_NGS_assembly TGGAGCATGTGATTATGGAGCAGGATCACATGGATTTTCCCGGCATTTGA 7100

**************************************************

Ctg654_12_Sangerassembly ACGAGCATAACACAGTGAAGCAGTACACATTACATAAACTGGGGGGGAAG 7043

Ctg654_12_NGS_assembly ACGAGCATAACACAGTGAAGCAGTACACATTACATAAACTGGGGGGGAAG 7150

**************************************************

Ctg654_12_Sangerassembly TAAAATGTCAAGTTATTGATAACCTAATTATCTGATGTTTTGAGCTCCTT 7093

Ctg654_12_NGS_assembly TAAAATGTCAAGTTATTGATAACCTAATTATCTGATGTTTTGAGCTCCTT 7200

**************************************************

Ctg654_12_Sangerassembly TGGGTTTGTTTGTGTGACCTTCACTTTTTGTATTTCTCACTTAACCTGAT 7143

Ctg654_12_NGS_assembly TGGGTTTGTTTGTGTGACCTTCACTTTTTGTATTTCTCACTTAACCTGAT 7250

**************************************************

Ctg654_12_Sangerassembly GTTTTTTCATTTTTCAATCCATGAACCACCGTTAAATGTGTGTGTATTTT 7193

Ctg654_12_NGS_assembly GTTTTTTCATTTTTCAATCCATGAACCACCGTTAAATGTGTGTGTATTTT 7300

**************************************************

Ctg654_12_Sangerassembly TTTTCTCCAGGCAAAAGAGATCCTGACCAAGGAGTCAAACGTCCAGGAGG 7243

Ctg654_12_NGS_assembly TTTTCTCCAGGCAAAAGAGATCCTGACCAAGGAGTCAAACGTCCAGGAGG 7350

**************************************************

Ctg654_12_Sangerassembly TGAGATGTCCGGTGACAGTGTGCGGTGACGTGCATGGCCAGTTCCATGAC 7293

Ctg654_12_NGS_assembly TGAGATGTCCGGTGACAGTGTGCGGTGACGTGCATGGCCAGTTCCATGAC 7400

**************************************************

Ctg654_12_Sangerassembly CTCATGGAGCTGTTCAAGATCGGCGGGAAATCACCAGACACAAACTATTT 7343

Ctg654_12_NGS_assembly CTCATGGAGCTGTTCAAGATCGGCGGGAAATCACCAGACACAAACTATTT 7450

**************************************************

Ctg654_12_Sangerassembly GTTTATGGGAGATTACGTGGACAGAGGGTACTACTCAGTAGAAACCGTCA 7393

Ctg654_12_NGS_assembly GTTTATGGGAGATTACGTGGACAGAGGGTACTACTCAGTAGAAACCGTCA 7500

**************************************************

Ctg654_12_Sangerassembly CTTTACTAGTAGCACTTAAGGTGAGTAATAATTTTAAAAAGTCCTCACAC 7443

Ctg654_12_NGS_assembly CTTTACTAGTAGCACTTAAGGTGAGTAATAATTTTAAAAAGTCCTCACAC 7550

**************************************************

Ctg654_12_Sangerassembly ACTTTGCTCGCTCCAAAACTGAAATTTGATAGGGCTTTTTAGGAAAAAAA 7493

Ctg654_12_NGS_assembly ACTTTGCTCGCTCCAAAACTGAAATTTGATAGGGCTTTTTAGGAAAAAAA 7600

**************************************************

Ctg654_12_Sangerassembly AATTAAGACACAGCTTTGCTCTCTCATATCATGTTTCAGGTACGCTTCCG 7543

Ctg654_12_NGS_assembly AATTAAGACACAGCTTTGCTCTCTCATATCATGTTTCAGGTACGCTTCCG 7650

**************************************************

Ctg654_12_Sangerassembly GGAGCGCATCACAATCCTCAGGGGGAACCACGAGAGCAGACAGATCACAC 7593

Ctg654_12_NGS_assembly GGAGCGCATCACAATCCTCAGGGGGAACCACGAGAGCAGACAGATCACAC 7700

**************************************************

Ctg654_12_Sangerassembly AAGTGTACGGCTTCTATGACGAGTGCCTCAGGAAATATGGTAACGCCAAT 7643

Ctg654_12_NGS_assembly AAGTGTACGGCTTCTATGACGAGTGCCTCAGGAAATATGGTAACGCCAAT 7750

**************************************************

Ctg654_12_Sangerassembly GTGTGGAAGTACTTCACAGACCTGTTCGATTACCTCCCCCTCACTGCCTT 7693

Ctg654_12_NGS_assembly GTGTGGAAGTACTTCACAGACCTGTTCGATTACCTCCCCCTCACTGCCTT 7800

**************************************************

Ctg654_12_Sangerassembly GGTAGACTCTCAGGTGAGAAAACACGTTGGTTAGTTTCACAGCTATAAAT 7743

Ctg654_12_NGS_assembly GGTAGACTCTCAGGTGAGAAAACACGTTGGTTAGTTTCACAGCTATAAAT 7850

**************************************************

Ctg654_12_Sangerassembly ATGGCTTGTGCAGAGACTGAGCAGGATTGAAAATGTGGAACAGCACATAC 7793

Ctg654_12_NGS_assembly ATGGCTTGTGCAGAGACTGAGCAGGATTGAAAATGTGGAACAGCACATAC 7900

**************************************************

Ctg654_12_Sangerassembly AGCATTATTAGTTGAGGAAGTGTTGATTAGATGGTAAGAGCATGCAGTGG 7843

Ctg654_12_NGS_assembly AGCATTATTAGTTGAGGAAGTGTTGATTAGATGGTAAGAGCATGCAGTGG 7950

**************************************************

Ctg654_12_Sangerassembly ATTCAGAGGCTGATGGTCATGAGTGAAGTACTGAAAGTTTCTTCTTTTCC 7893

Ctg654_12_NGS_assembly ATTCAGAGGCTGATGGTCATGAGTGAAGTACTGAAAGTTTCTTCTTTTCC 8000

**************************************************

Ctg654_12_Sangerassembly TTCCAGATTTTCTGCCTTCATGGAGGCCTGTCACCGTCCATAGACACATT 7943

Ctg654_12_NGS_assembly TTCCAGATTTTCTGCCTTCATGGAGGCCTGTCACCGTCCATAGACACATT 8050

**************************************************

Ctg654_12_Sangerassembly GGATCACATTAGAGCACTGGACCGTTTACAGGAAGTGCCACATGAGGTAA 7993

Ctg654_12_NGS_assembly GGATCACATTAGAGCACTGGACCGTTTACAGGAAGTGCCACATGAGGTAA 8100

**************************************************

Ctg654_12_Sangerassembly AGATAATACGTACAAGACAACAAAGAACTGAACAGAAAACAACAGGATTT 8043

Ctg654_12_NGS_assembly AGATAATACGTACAAGACAACAAAGAACTGAACAGAAAACAACAGGATTT 8150

**************************************************

Ctg654_12_Sangerassembly GGTTGTAAAATAATAGCCATCAATAAAATCAAGTTGATCATGACCAATAA 8093

Ctg654_12_NGS_assembly GGTTGTAAAATAATAGCCATCAATAAAATCAAGTTGATCATGACCAATAA 8200

**************************************************

Ctg654_12_Sangerassembly CTAGAAAGCACAAAGGAAGTAATGTGTTGATATTCTCATTTCTCCTTAAT 8143

Ctg654_12_NGS_assembly CTAGAAAGCACAAAGGAAGTAATGTGTTGATATTCTCATTTCTCCTTAAT 8250

**************************************************

Ctg654_12_Sangerassembly TCAGTATATTTGCAGTTAAAACATGAGGGCGGCTGTTTAGGTAAGCAACA 8193

Ctg654_12_NGS_assembly TCAGTATATTTGCAGTTAAAACATGAGGGCGGCTGTTTAGGTAAGCAACA 8300

**************************************************

Ctg654_12_Sangerassembly GCAGATCTGCTTCTGAGTCCCATCACTAACATTCTCCTCCTCCTCCTATT 8243

Ctg654_12_NGS_assembly GCAGATCTGCTTCTGAGTCCCATCACTAACATTCTCCTCCTCCTCCTATT 8350

**************************************************

Ctg654_12_Sangerassembly TGTCCTCCACAGGGTCCCATGTGTGACCTGCTGTGGTCAGACCCCGACGA 8293

Ctg654_12_NGS_assembly TGTCCTCCACAGGGTCCCATGTGTGACCTGCTGTGGTCAGACCCCGACGA 8400

**************************************************

Ctg654_12_Sangerassembly CCGTGGCGGCTGGGGCATCTCTCCTCGAGGAGCCGGCTACACTTTCGGTC 8343

Ctg654_12_NGS_assembly CCGTGGCGGCTGGGGCATCTCTCCTCGAGGAGCCGGCTACACTTTCGGTC 8450

**************************************************

Ctg654_12_Sangerassembly AGGACATCTCAGAGACTTTCAACCACGCCAACCGCCTCACACTGGTGTCC 8393

Ctg654_12_NGS_assembly AGGACATCTCAGAGACTTTCAACCACGCCAACCGCCTCACACTGGTGTCC 8500

**************************************************

Ctg654_12_Sangerassembly CGTGCCCACCAGCTGGTTATGGAGGTGTGTGTCTGTTAAGATTGTGTTTT 8443

Ctg654_12_NGS_assembly CGTGCCCACCAGCTGGTTATGGAGGTGTGTGTCTGTTAAGATTGTGTTTT 8550

**************************************************

Ctg654_12_Sangerassembly TTTTTTGTGAGTCCAGTACAGTAAAGTAGATTTATATCAACTGGCCACAG 8493

Ctg654_12_NGS_assembly TTTTTTGTGAGTCCAGTACAGTAAAGTAGATTTATATCAACTGGCCACAG 8600

**************************************************

Ctg654_12_Sangerassembly TGGCTGAGCTGAATAATCGTTGGTTATGTGACTAAGTGGCTGTTACAGTT 8543

Ctg654_12_NGS_assembly TGGCTGAGCTGAATAATCGTTGGTTATGTGACTAAGTGGCTGTTACAGTT 8650

**************************************************

Ctg654_12_Sangerassembly TATAGACTACTCAGCTATGGTCAAAAACCACCACTACAGAAAACAAAAGG 8593

Ctg654_12_NGS_assembly TATAGACTACTCAGCTATGGTCAAAAACCACCACTACAGAAAACAAAAGG 8700

**************************************************

Ctg654_12_Sangerassembly AGAGTTATAACACCACCAGCCAGCTCCCTTGTACCAAGTTCAAGAACATG 8643

Ctg654_12_NGS_assembly AGAGTTATAACACCACCAGCCAGCTCCCTTGTACCAAGTTCAAGAACATG 8750

**************************************************

Ctg654_12_Sangerassembly CTGTGCAGTTTAGATTTGCATTATTGTGGCACTTTTTCCTGAATTATACA 8693

Ctg654_12_NGS_assembly CTGTGCAGTTTAGATTTGCATTATTGTGGCACTTTTTCCTGAATTATACA 8800

**************************************************

Ctg654_12_Sangerassembly TCTGCAGTGAATAATGGATTGCATCTGCTTCCCCCAGGGTTACAACTGGT 8743

Ctg654_12_NGS_assembly TCTGCAGTGAATAATGGATTGCATCTGCTTCCCCCAGGGTTACAACTGGT 8850

**************************************************

Ctg654_12_Sangerassembly GCCATGAGAGGAATGTGGTGACAATATTTAGCGCTCCCAACTACTGCTAC 8793

Ctg654_12_NGS_assembly GCCATGAGAGGAATGTGGTGACAATATTTAGCGCTCCCAACTACTGCTAC 8900

**************************************************

Ctg654_12_Sangerassembly CGCTGTGGCAACCAAGCGGCTATCATGGAACTAGATGACACTCTCAAATA 8843

Ctg654_12_NGS_assembly CGCTGTGGCAACCAAGCGGCTATCATGGAACTAGATGACACTCTCAAATA 8950

**************************************************

Ctg654_12_Sangerassembly CTCATTGTAAGTATATTTAAAAAAATGTAAAATAATTCTATAGACTTATA 8893

Ctg654_12_NGS_assembly CTCATTGTAAGTATATTTAAAAAAATGTAAAATAATTCTATAGACTTATA 9000

**************************************************

Ctg654_12_Sangerassembly AAAGTTAATGCTCTGCATGACTGGGAGAGTATTTTTTCCATGAGATAGGG 8943

Ctg654_12_NGS_assembly AAAGTTAATGCTCTGCATGACTGGGAGAGTATTTTTTCCATGAGATAGGG 9050

**************************************************

Ctg654_12_Sangerassembly CTACAGGCTTTTTCAAAATTCAAATTATGTGCAATATATTAATATAAATG 8993

Ctg654_12_NGS_assembly CTACAGGCTTTTTCAAAATTCAAATTATGTGCAATATATTAATATAAATG 9100

**************************************************

Ctg654_12_Sangerassembly GATACATGTATTACTTGAATTTGGTCAGCACATCTAATTAGTTTTTTTTA 9043

Ctg654_12_NGS_assembly GATACATGTATTACTTGAATTTGGTCAGCACATCTAATTAGTTTTTTTTA 9150

**************************************************

Ctg654_12_Sangerassembly CTGATTTTTATTTCTTCCTTTACCCAGCTTGCAGTTTGATCCTGCGCCTC 9093

Ctg654_12_NGS_assembly CTGATTTTTATTTCTTCCTTTACCCAGCTTGCAGTTTGATCCTGCGCCTC 9200

**************************************************

Ctg654_12_Sangerassembly GCAGAGGGGAGCCTCACGTCACCCGTCGCACCCCAGACTACTTCC----- 9138

Ctg654_12_NGS_assembly GCAGAGGGGAGCCTCACGTCACCCGTCGCACCCCAGACTACTTCCTGTAA 9250

*********************************************

**Supplementary information 5:** All the cDNA sequences of 11 genes with potential growth function for the differential expression studies between three tissues of fast- and slow-growing Asian seabass (**see the separate FASTA file)**

**Supplementary information 6:** Available BAC end sequences from the 72 sequenced BAC clones **(see the separate FASTA file)**
